# Extracellular domain 2 of TSPAN4 governs its functions

**DOI:** 10.1016/j.bpr.2024.100149

**Published:** 2024-03-05

**Authors:** Raviv Dharan, Alisa Vaknin, Raya Sorkin

**Affiliations:** 1 School of Chemistry, Raymond & Beverly Sackler Faculty of Exact Sciences, Tel Aviv University, Tel Aviv, Israel; 2 Center for Physics and Chemistry of Living Systems, Tel Aviv University, Tel Aviv, Israel

**Keywords:** tetraspanin, curvature sensitivity, micropipette aspiration, optical tweezers

## Abstract

Tetraspanin 4, a protein with four transmembrane helices and three connecting loops, senses membrane curvature and localizes to membrane tubes. This enrichment in tubular membranes enhances its diverse interactions. While the transmembrane part of the protein likely contributes to curvature sensitivity, the possible roles of the ectodomains in curvature sensitivity of tetraspanin 4 are still unknown. Here, using micropipette aspiration combined with confocal microscopy and optical tweezers, we show that the extracellular loop 2 contributes to the curvature sensitivity and curvature-induced interactions of tetraspanin 4. To this end, we created truncated tetraspanin 4 mutants by deleting each of the connecting loops. Subsequently, we pulled membrane tubes from giant plasma membrane vesicles containing tetraspanin 4-GFP or its mutants while maintaining controllable membrane tension and curvature. Among the mutations tested, the removal of the extracellular loop 2 had the most significant impact on both the curvature sensitivity and interactions of tetraspanin 4. Based on the results, we suggest that the extracellular loop 2 regulates the affinity of tetraspanin 4 towards curved membranes and affects its lateral interactions.

## Why it matters

Tetraspanins are widespread in nearly every cell, showcasing diverse functions linked to crucial cellular and pathological processes like cell adhesion, immune signaling, cell-cell fusion, viral infection, and cancer metastasis. This underscores their significance in cellular mechanisms and suggests potential therapeutic applications. Their various cellular roles are closely tied to their ability to form higher-order structures. The assembly of tetraspanins is likely dependent on their membrane concentration, which increases in curved membranes for some tetraspanins due to their membrane curvature sensitivity. Elucidating the molecular domains governing their curvature sensitivity and interactions is essential for understanding tetraspanin dynamics in the membrane. Here, we demonstrate that the extracellular 2 loop of tetraspanin 4 is crucial for both curvature sensitivity and domain formation, suggesting a way to regulate tetraspanin function.

## Main text

**Figure 1:**
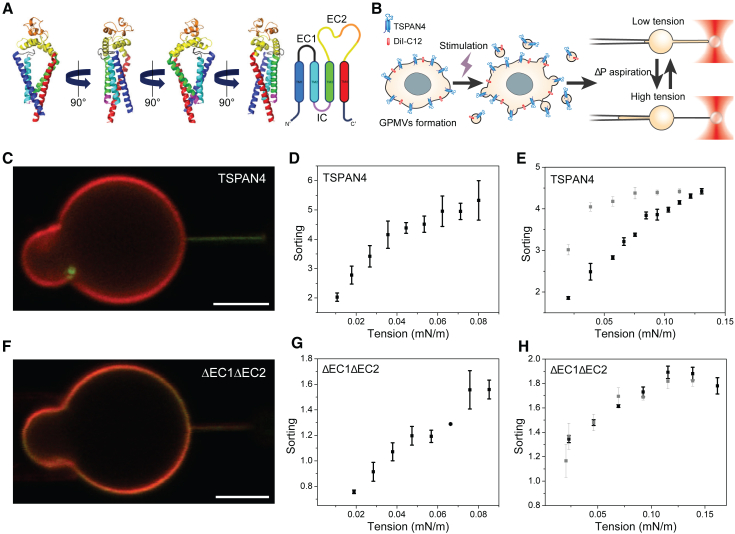
Deletion of EC1 and EC2 reduces TSPAN4 sorting in curved membranes. (*A*) Tetraspanin 4 (TSPAN4)-AlphaFold structure prediction illustration. Each structural domain is shown in a different color: TM1, blue; EC1, black; TM2, cyan; IC, magenta; TM3, green; EC2, yellow; and small loop of the EC2, orange. On the right: schematic representation of TSPAN4 structure. (*B*) Illustration of the experimental procedure: formation of giant plasma membrane vesicles (GPMVs) containing TSPAN4 and DiI-C12, followed by a membrane tube-pulling assay. (*C*) Confocal microscopy of a GPMV containing WT TSPAN4-GFP (green) and DiI-C12 (red). (*D*) TSPAN4 sorting as a function of membrane tension plot showing that TSPAN4 sorting increases with membrane tension. (*E*) TSPAN4 sorting as function of membrane tension showing sorting increase with membrane tension followed by sorting hysteresis upon tension decrease. Black squares represent tension increase, whereas gray squares represent tension decrease. (*F*) Confocal microscopy of a GPMV containing TSPAN4ΔEC1ΔEC2-GFP and DiI-C12. (*G* and *H*) TSPAN4ΔEC1ΔEC2 sorting as a function of membrane tension plots. Black squares represent tension increase, whereas gray squares represent tension decrease. All scale bars represent 5 μm. Error bars are SEM. (*C*)–(*E*) are adapted from (14).

The tetraspanin family of proteins contains 33 known members in humans that are highly conserved across species (1,2). They participate in numerous cellular processes including adhesion, migration, signaling, fusion, fission, and immune cell functions (3,4,5,6,7). Tetraspanins are known to interact with themselves and other cell surface receptors like integrins, forming tetraspanin-enriched microdomains (8,9,10). One of the tetraspanins that was found to form membrane domains of various sizes is tetraspanin 4 (TSPAN4) (11,12). TSPAN4-enriched domains were found to regulate cellular processes like migrasome formation and cell membrane repair (11,12,13). Like all tetraspanins, TSPAN4 spans the cell membrane with four transmembrane helices (TM1–TM4), which are connected by extracellular and intracellular (IC) loops (Fig. 1 *A*). Extracellular loop 1 (EC1), connecting TM1 and TM2, contains amino acids 39–51. The IC loop strictly connects TM2 and TM3 due to its small length that encompasses amino acids 78–84. EC2, connecting TM3 and TM4, is relatively large and contains amino acids 109–199.

Previously, we demonstrated that TSPAN4 is sensitive to membrane curvature and undergoes significant enrichment in membranes with positive membrane curvature (14). We generated giant plasma membrane vesicles (GPMVs) from HEK293T cells expressing TSPAN4-GFP and labeled them with the plasma membrane dye DiI-C12 (Fig. 1 *B*). We used HEK293T cells since their endogenous TSPAN4 levels are very low compared to TSPAN4-transfected HEK293T cells, as previously demonstrated by mass spectrometry (12). The membrane dye DiI-C12 was chosen after we examined different membrane dyes for labeling GPMVs (14). By integrating micropipette aspiration with optical tweezers, we pulled membrane tubes from aspirated GPMVs (Fig. 1 *B*). By setting the aspiration pressure in the pipette, we regulated the membrane tension of the vesicle and thus the membrane curvature of the tube (15,16). Using the fluorescence signal of the labeled protein and the lipid dye, we quantified the sorting ratio (17,18):

$$S=\frac{{\left(I_{TSPAN4-GFP}/I_{DiI-C12}\right)}_{tube}}{{\left(I_{TSPAN4-GFP}/I_{DiI-C12}\right)}_{GPMV}}\text{,}$$

where I is the fluorescence intensity of GFP or DiI-C12. These measurements revealed that TSPAN4 is progressively sorted into the tube with increasing membrane tension (Fig. 1, *C* and *D*). As TSPAN4 is known to interact with itself and other proteins, the enrichment of TSPAN4 in the tube increased its probability for interactions. To investigate whether TSPAN4 interactions affect the redistribution of TSPAN4, we measured the sorting upon gradual tension increase followed by gradual decrease in the tension (Figs. 1 *B* and S1) and discovered that the sorting exhibited hysteresis (Fig. 1 *E*). Furthermore, we demonstrated that shaving of the extracellular loops of TSPAN4 in the GPMVs resulted in the abolishment of the sorting hysteresis, which suggests that the ECs mediate the curvature-induced TSPAN4 interactions (14).

Here, we set out to uncover the functionality of the ECs of TSPAN4 by implementing the assay described above. We deleted EC1 and EC2 and expressed the new protein TSPAN4ΔEC1ΔEC2-GFP in HEK293T cells. Following GPMV formation, the protein was observed in the membrane of the GPMVs (Fig. S2). Using the tube-pulling assay, we found that the deletion of EC1 and EC2 significantly reduced the curvature sensitivity of TSPAN4 (Fig. 1, *F* and *G*). The membrane tube is significantly less enriched with TSPAN4ΔEC1ΔEC2-GPF, as seen from its low green fluorescence intensity (Fig. 1 *F*), compared with the wild-type (WT) TSPAN4 tube fluorescence (Fig. 1 *C*). The sorting values at relatively low membrane tensions, which correspond to tubes with relatively large radii (or low membrane curvature), were approximately one or even lower, indicating that the protein density in the tube was equal to or lower than its density in the flat membrane of the vesicle. When the membrane tension was raised, the sorting of TSPAN4ΔEC1ΔEC2 increased but was still significantly lower compared to the WT-TSPAN4, even at very high membrane curvature (Figs. 1 *G* and S3). Nevertheless, the protein density within tubes exhibiting high membrane curvature was approximately twice as high as that in the flat vesicle, indicating the inherent affinity of TSPAN4 for curved membranes. When the membrane tension was decreased, sorting hysteresis was not observed (Fig. 1 *H*).

When the ECs were shaved in our previous work (14), TSPAN4 curvature sensitivity was not affected. This difference between the results can be explained by the fact that in the WT-TSPAN4 experiments, the shaving of the loops occurred after the protein was embedded in the membrane, whereas in the TSPAN4ΔEC1ΔEC2, the deletion occurred before the insertion of the protein into the membrane. The protein segments likely to have the greatest impact on the curvature sensitivity of transmembrane proteins are the helices located inside the membrane (19,20,21), i.e., TM1–TM4 for TSPAN proteins. The shaving of the loops following natural insertion into the membrane, with the correct configuration of the transmembrane helices, probably did not significantly alter their orientations, and thereby the sorting was not affected. In the TSPAN4ΔEC1ΔEC2 mutant, the loops that connected TM1–TM2 and TM3–TM4 were absent during the protein synthesis, and thereby the orientations of these TMs were probably altered compared to the WT-TSPAN4.

**Figure 2:**
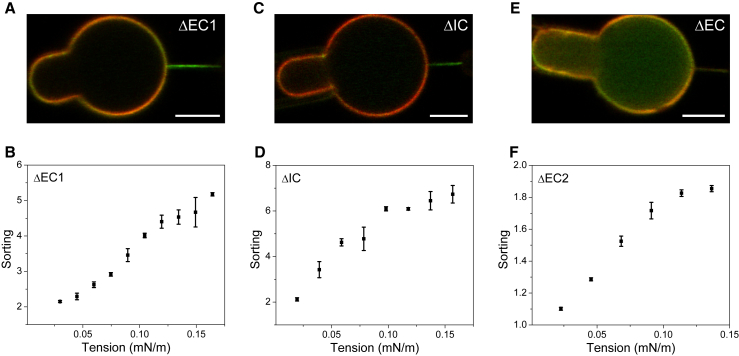
Curvature sensitivity of TSPAN4-loop-deletion mutants. (*A*–*C*) Confocal microscopy images of membrane tubes pulled from aspirated GPMVs dyed with DiI-C12 and containing TSPAN4ΔEC1 (A), TSPAN4ΔIC (B), or TSPAN4ΔEC2 (C). Scale bars represent 5 μm. (*D*–*F*) Sorting ratio as a function of membrane tension plots of each mutant as indicated. Error bars are SEM.

**Figure 3:**
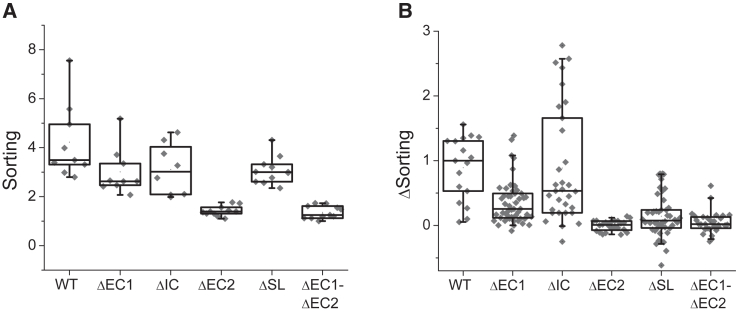
Curvature sensitivity and sorting hysteresis of TSPAN4 mutants. (*A*) Boxplot comparing the sorting values of TSPAN4 (WT), TSPAN4ΔEC1, TSPAN4ΔIC, TSPAN4ΔEC2, TSPAN4ΔSL, and TSPAN4ΔEC1ΔEC2 obtained from tube-pulling experiments conducted at membrane tension of 0.06±0.005mN/m. From left to right: n = 9, 9, 8, 11, 11, and 10 GPMVs. (*B*) Boxplot comparing the sorting ratio difference between the tension decrease and tension increase paths for the different TSPAN4 mutants. From left to right: n = 3, 9, 5, 6, 10, and 5 GPMVs. Box-whisker plot horizontal lines represent (from bottom to top) the 5%, 25%, 50%, 75%, and 95% of the data.

In order to examine the impact of each loop connecting the four transmembrane helices on TSPAN4 sorting, we deleted each loop separately. First, we deleted the EC1 from the TSPAN4 and repeated the tube-pulling experiment. The deletion did not significantly affect the sorting of TSPAN4 into the membrane tubes (Fig. 2 *A*). Sorting was evident even at relatively low membrane tension and substantially increased with further tension increase (Figs. 2 *B* and S4). Next, we deleted the IC loop, which connects the second and third transmembrane helices of TSPAN4. Similarly to the EC1 deletion results, the deletion of IC loop did not affect the tubular enrichment of TSPAN4 (Fig. 3 *C*), and the sorting ratio reached high values (Figs. 2 *D* and S5). We next deleted the EC2 from the protein. This deletion drastically affected TSPAN4 membrane distribution and reduced its tubular enrichment (Fig. 2 *E*). Similarly to the TSPAN4ΔEC1ΔEC2 results, the sorting values at low tension were close to one (Figs. 2 *F* and S6) and increased to around two at high membrane tension. Fig. 3 *A* shows the sorting values of the different TSPAN4 mutants obtained from experiments conducted under the same membrane tension. The results show that the deletion of EC2, compared to the deletion of EC1 and IC, significantly reduced the sorting of TSPAN4 into curved membranes. The reduction in the curvature sensitivity for TSPAN4ΔEC2 implies that the EC2 contributes to the conical structure of TSPAN4. The most variable region among different TSPANs is the EC2 domain, and it is associated with the diverse interactions observed among various TSPANs (22,23). Our results suggest that the EC2 may also be crucial for the curvature sensitivity of TSPANs.

**Figure 4:**
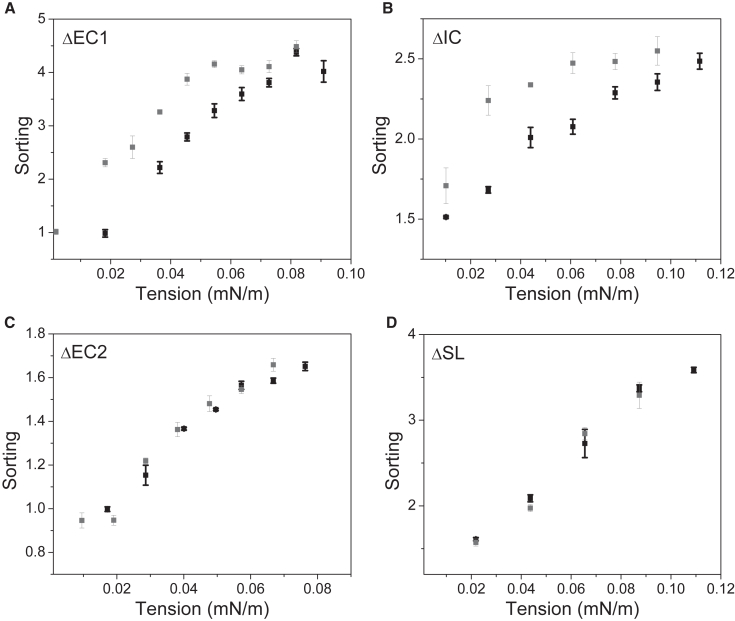
EC2 deletion abolished TSPAN4 sorting hysteresis. (*A*–*D*) Sorting as a function of the membrane tension plots of TPSAN4ΔEC1 (*A*), TSPAN4ΔIC (*B*), TSPAN4ΔEC2 (*C*), and TSPAN4ΔSL (*D*). In all images, black squares represent tension increase, whereas gray squares represent tension decrease. Error bars are SEM.

Next, we wanted to examine the sorting hysteresis of TSAPN4 with the truncated mutants that we created. The sorting values for increasing tension were evaluated for each of the mutants and compared to sorting values observed upon tension decrease (Fig. 3 *B*). Like the curvature sensitivity results, TSPAN4ΔEC1 and TSPAN4ΔIC showed hysteresis in the sorting values, whereas TSPAN4ΔEC2 did not (Figs. 3 *B* and 4, *A–C*). The sorting hysteresis of TSPAN4ΔEC1 was lower compared to WT-TSPAN4 (Figs. 3 *B*, 4 *A*, and S7), implying that the interactions were likely impaired for this mutant. Deletion of IC did not significantly affect the sorting hysteresis of TSPAN4 (Figs. 3 *B*, 4 *B*, and S8). The sorting hysteresis of TSPAN4ΔEC2, however, was completely abolished (Figs. 3 *B*, 4 *C*, and S9), suggesting that EC2 is the dominant part that mediates TSAPN4 curvature-induced interactions. The reduced interactions of TSPAN4ΔEC2 can result from the reduced protein enrichment in the tube and not only from the absence of EC2.

To further explore how EC2 contributes to TSPAN4 curvature-induced interactions, we created a new mutant with deletion of a small loop (SL) from the EC2 domain. The SL, containing amino acids 151–187, is one of the main structural differences between CD9 and TSPAN4 according to the CD9 crystal structure and AlphaFold prediction of TSPAN4 (24,25,26). The alignment of the proteins shows that all other regions, including the transmembrane helices, have almost identical configurations (Fig. S10) and thereby probably the same curvature affinity. Our previous results (14) showed that, indeed, the proteins have the same intrinsic curvature; however, they differ in the sorting hysteresis, suggesting that the SL contributes to TSPAN4 interactions. TSPAN4ΔSL was sensitive to membrane curvature and exhibited high enrichment in the tube (Figs. 3 *B*, 4 *D*, and S11). The sorting hysteresis, however, was significantly reduced (Figs. 3 B, 4 *D*, and S12). The fact that the SL deletion affects the sorting hysteresis and not the curvature sensitivity suggests that the absence of the EC2 in TSPAN4ΔEC2 was the leading reason for the lack of curvature-induced interactions rather than the reduced tube enrichment. It is important to note that the interactions mediated by the EC2, leading to the sorting hysteresis, could involve TSPAN4 self-interactions, interactions with other TSPANs, and/or interactions with other types of proteins (8,27,28).

Overall, we investigated each of the loops that connect the four TMs of TSPAN4. Our results show that the EC1 and IC loops do not influence the curvature sensitivity of TSPAN4. Furthermore, the IC loop does not affect TSPAN4 curvature-induced interactions, whereas EC1 seems to be involved in interactions to some extent. Notably, the EC2 was crucial for TSPAN4 curvature-induced interactions. Likewise, it contributes to the curvature sensitivity of the protein probably by allowing the correct conical configuration of the TMs. Although the curvature sensitivity was significantly damaged in TSPAN4ΔEC2 and TSPAN4ΔEC1EΔC2, the protein was still enriched in the highly curved membrane, likely due to its intrinsic conical structure.

TSPAN proteins are crucial for essential cellular processes including fertilization and migrasome formation (12,29). TSPAN4 was found to become enriched in curved membranes and to associate into membrane domains which are crucial for cellular processes like migrasome formation (11). The EC2 protein ectodomain includes a cysteine-cysteine-glycine motif and cysteine residues, which are thought to be essential for efficient intercellular interactions between TSPANs and other associated proteins (30,31). As TSPANs’ mode of action is highly linked to their molecular interactions and membrane domain formation, the EC2 domain might be a regulator of TSPAN proteins that determines their level of enrichment in curved membranes and governs their interactions with other molecules. Hence, it is plausible that the EC2 domain serves as crucial region for modulating the biological functions of TSPANs.

## Author contributions

R.D., A.V., and R.S. designated the research; R.D. and A.V. performed the experiments; R.D. analyzed data; and R.D. and R.S. wrote the manuscript.

## Supporting material

Document S1. Supporting materials and methods and Figures S1–S12

Document S2. Article plus supporting material
