# Supplementary material for: Extracellular domain 2 of TSPAN4 governs its functions
Source: Biophys Rep (N Y). 2024 Mar 5;4(2):100149. doi: 10.1016/j.bpr.2024.100149 (PMC10982557; doi:10.1016/j.bpr.2024.100149)
Supplement: Document S1. Supporting materials and methods and Figures S1–S12 [file mmc1.pdf]

BPR, Volume *4*

Supplemental information

Extracellular domain 2 of TSPAN4 governs its functions

Raviv Dharan, Alisa Vaknin, and Raya Sorkin

Supplementary material for

Extracellular domain 2 of TSPAN4 governs its functions

Raviv Dharan[1,2], Alisa Vaknin[1,2] and Raya Sorkin*[1,2]

1. School of Chemistry, Raymond & Beverly Sackler Faculty of Exact Sciences, Tel Aviv University, Israel
2. Center for Physics and Chemistry of Living Systems, Tel Aviv University, Tel Aviv, Israel

*Corresponding author: Raya Sorkin, Email: rsorkin@tauex.tau.ac.il

Materials and methods

TSPAN4 mutants expression plasmid preparation.

The complementary DNA of mouse-TSPAN4 was sub-cloned into the pEGFP-N1 vector (Addgene). To generate the following truncated versions of TSPAN4 (UniProt accession number Q9DCK3): deletion of the amino acids 39-51 (TSPAN4ΔEC1), 78-84 (TSPAN4ΔIC), 109-199 (TSPAN4ΔEC2), 39-51 and 109-199 (TSPAN4ΔEC1ΔEC2), or 151-187 (TSPAN4ΔSL), we applied the reverse PCR approach. First, a PCR was performed, generating a linear vector using the following primers:

Construct name	Primers (5' → 3')
TSPAN4ΔEC1	Forward: TCGGCTGCCAACCTGCTCA<br>Reverse: CTGTGTGGCAGCCAACCAGATGC
TSPAN4ΔEC2	Forward: GAGAACCTGCTAGCTGTGGGCATCTTTGGA<br>Reverse: ACTGTAGGCAAAGAAGAGCACAGCAATGG
TSPAN4ΔEC1ΔEC2	Same as for TSPAN4ΔEC1 and TSPAN4ΔEC2 template
TSPAN4ΔIC	Forward: ACTTTCTTTGTGCTGCTGCTGCTAGTGTT<br>Reverse: CTTGAGGGCCCCAATGCAGCCCA
TSPAN4ΔSL	Forward: TCGCCCTGTTATGAGACAGTGAAGGCC<br>Reverse: AACTCCACAGCATCGGAAATCAGTCTGGAT

The product was applied to DpnI (NEB, R0176) residual methylated DNA digestion, followed by purification using the NucleoSpin Gel and PCR-Clean-up (Macherey-Nagel 740609). Next, the linear vector was phosphorylated using the T4 polynucleotide kinase (NEB, M0201) at 37 °C for 30 minutes and followed by a deactivation step for 20 minutes at 65 °C. Subsequently, the phosphorylated product was used for a ligation reaction with T4 DNA ligase (NEB, M0202) for 2 hours at room temperature. Finally, 5 µL of the ligated product was transformed into chemically competent DH5α *Escherichia coli* cells. A colony was inoculated, followed by

plasmid extraction (Macherey-Nagel, 740727), and sent to Sanger sequencing (ZABAM Instrumentation and Service sequencing unit at Tel Aviv University).

Cell transfection and GPMV formation

HEK293T (ATCC CRL-3216^{TM}) cells were cultured at 37°C and 5% CO_2 in DMEM (Gibco-Thermo Fisher scientific 11995065) supplemented with 10% Fetal bovine serum (biological industries, 04-001-1A) and 1% penicillin-streptomycin (Gibco-Thermo Fisher scientific 15140122). 24 hours before cell transfection, the cells were plated in DMEM supplemented with 10% Fetal bovine serum at 20% confluency in a 25 cm^2 flask (Romical) coated with poly-L-lysine (Sigma-Aldrich P6282) to keep the cells attached during the blebbing process and to minimize cell debris in solution. At 50% confluency, cells were transiently transfected with 5 µg DNA using Lipofectamine 2000 (Invitrogen, Thermo Fisher scientific) according to the manufacture's protocols and then grown 24 hours for protein expression. GPMVs were produced according to a published protocol[1]. Briefly, following TSPAN4-GFP (or the different TSPAN4 mutants) expression the cell membrane was stained with DiI-C12 membrane dye (Invitrogen, Thermo Fisher scientific D383), washed with GPMV buffer (10 mM HEPES, 150 mM NaCl, 2 mM CaCl2, pH 7.4) twice, and incubated with 1 mL of GPMV buffer containing 1.9 mM DTT (Sigma-Aldrich 1019777701) and 27.6 mM formaldehyde (Sigma-Aldrich F8775). Secreted GPMVs were then collected and isolated from the cells and immediately used for optical trapping experiments. In order to verify the presence of TSPAN4-GFP in the GPMV membrane, we scanned the GPMVs using 488 laser before each measurement.

Tube pulling from aspirated GPMVs

The experiments were performed using a C-trap® confocal fluorescence optical tweezers setup (LUMICKS, Amsterdam, the Netherlands) made of an inverted microscope based on a water-immersion objective (NA 1.2) together with a condenser top lens placed above the sample. The optical traps are generated by splitting a 10W 1064-nm laser into two orthogonally polarized, independently steerable optical traps. To steer the two traps, one coarse-positioning piezo stepper mirror and one accurate piezo mirror were used. Optical traps were used to capture polystyrene microbeads. The displacement of the trapped beads from the center of the trap was measured and converted into a force signal by back-focal plane interferometry of the condenser lens using two position-sensitive detectors. The samples were illuminated by a bright field 850-nm LED and imaged in

transmission onto a metal-oxide semiconductor (CMOS) camera. **Confocal fluorescence microscopy:** The C-Trap uses a 3 color, fiber-coupled laser with wavelengths 488, 561 and 638 nm for fluorescence excitation. Scanning was done using a fast tip/tilt piezo mirror. For confocal detection, the emitted fluorescence was descanned, separated from the excitation by a dichroic mirror, and filtered using an emission filters (Blue: 500-550 nm, Green: 575-625 nm and Red: 650-750 nm). Photons were counted using fiber-coupled single-photon counting modules. The multimode fibers serve as pinholes providing background rejection. For confocal imaging the 488 nm and 532 nm lasers were used for GFP and DiI-C12 excitation with 5% and 1% laser power respectively, 54.34 µW is the maximal laser power, and the emission detected in three channels (Blue, Green, Red).

Experimental chamber: PDMS walls were placed on the bottom cover slip (Thorlabs CG15KH1) and mounted onto an automated XY-stage. The GPMVs were added to the chamber and after about 15 minutes, a few drops of oil were put on the sample surface to prevent evaporation. A micropipette aspiration setup including micromanipulator (Sensapex) holding a micropipette with diameter of 5 µm (BioMedical instruments) connected to a Fluigent EZ-25 pump was integrated to our optical tweezers instrument. Before each experiment, the zero-suction pressure was found by aspirating a 3.43 µm polystyrene bead (Spherotech) into the pipette and reducing the suction pressure until the bead stopped moving. A membrane tube was pulled from aspirated GPMVs using beads trapped by the optical tweezers. First, a membrane tube was pulled at relatively low suction pressure (0.05-0.1 mbar). Then we gradually increased the suction pressure (usually by 0.05-0.1 mbar steps) until we reached values in the range of 0.3-0.7 mbar. In the experiments where the membrane tube was not ruptured during the tension increase, we gradually decreased the pressure stepwise, until reaching the zero pressure.

Data Analysis

Data acquisition was carried out using Bluelake, a commercial software from Lumicks. This software stores experimental data acquired with the C-trap in HDF5 files, which can be processed using Lumicks' Pylake python package. Images of the confocal scans were reconstituted from photon count per pixel data in the HDF5 files using Pylake. All data analysis was performed with custom-written Python scripts. Fluorescence intensity profiles were obtained from the images by averaging the photon count of the relevant fluorescent channel (Blue or Green) in the region of interest.

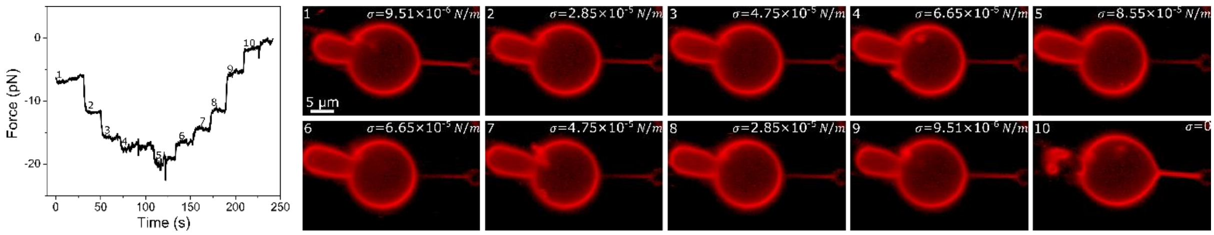

Figure S1. Tube pulling experiment- membrane tension increase followed by tension decrease. On the left, a plot showing the force to hold a tube as function of time during tension increase steps followed by subsequent tension decrease of aspirated GPMVs labelled with DiI-C12. The numbers indicate to the corresponding confocal images on the right presenting the values of the membrane tension applied by the micropipette aspiration. The image pixel intensities are presented in logarithmic scale. It can be seen that when the membrane tension was increased (1-5) the pulling force and the length of the tongue of the vesicle inside the pipette increased as well, whereas the radius of the tube decreased. When the membrane tension was decreased (6-10), the trends were reversed.

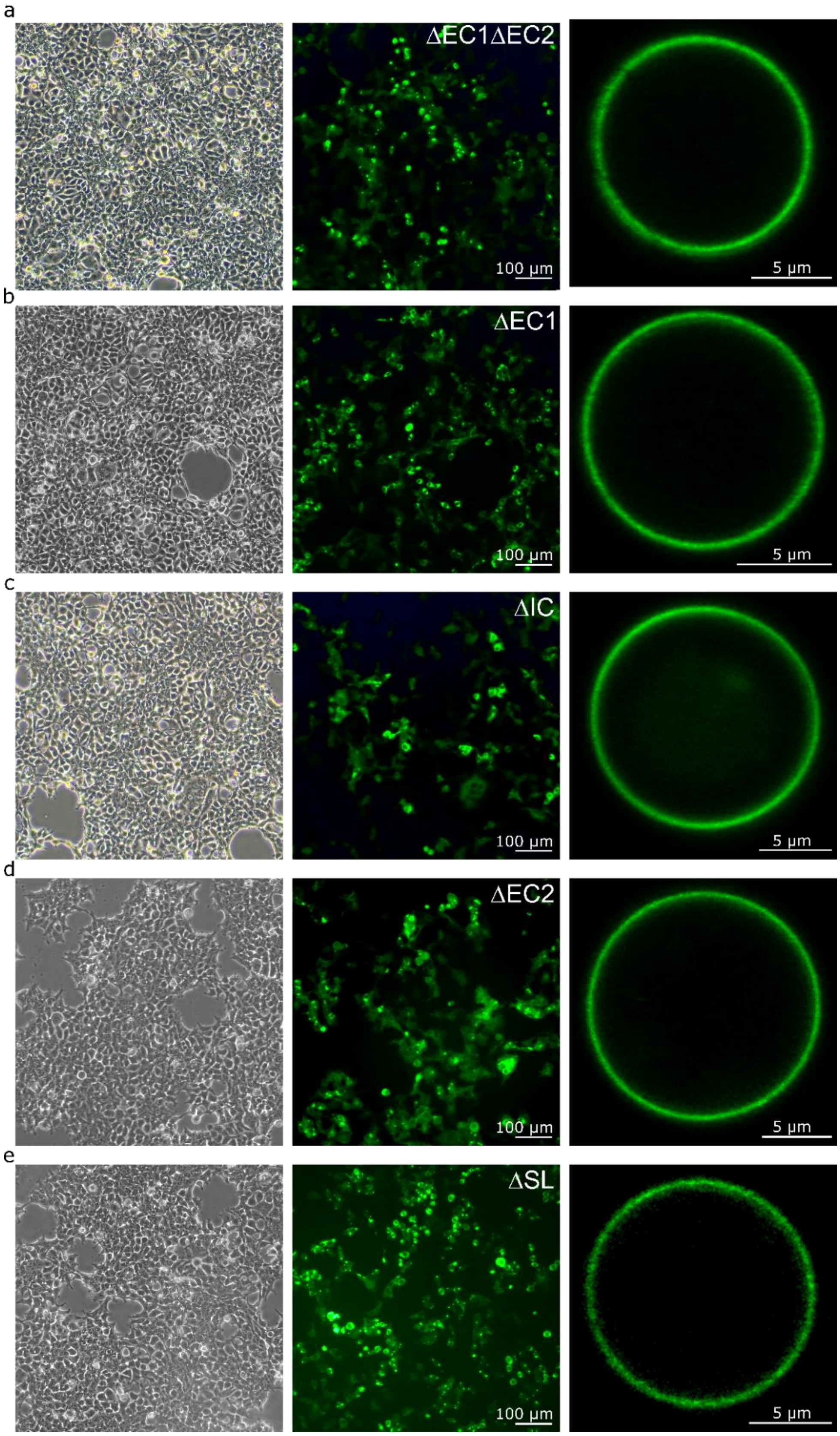

Figure S2. TSPAN4 mutants' expression. (a-e) Bright field (left) and green channel (middle) microscopy images of HEK293T cells expressing TSPAN4ΔEC1ΔEC2-GFP (a), TSPAN4ΔEC1-GFP (b), TSPAN4ΔIC-GFP (c), TSPAN4ΔEC2-GFP (d) and TSPAN4ΔSL-GFP (e). On the right, confocal microscopy images of isolated GPMVs containing TSPAN4 mutants in their membrane.

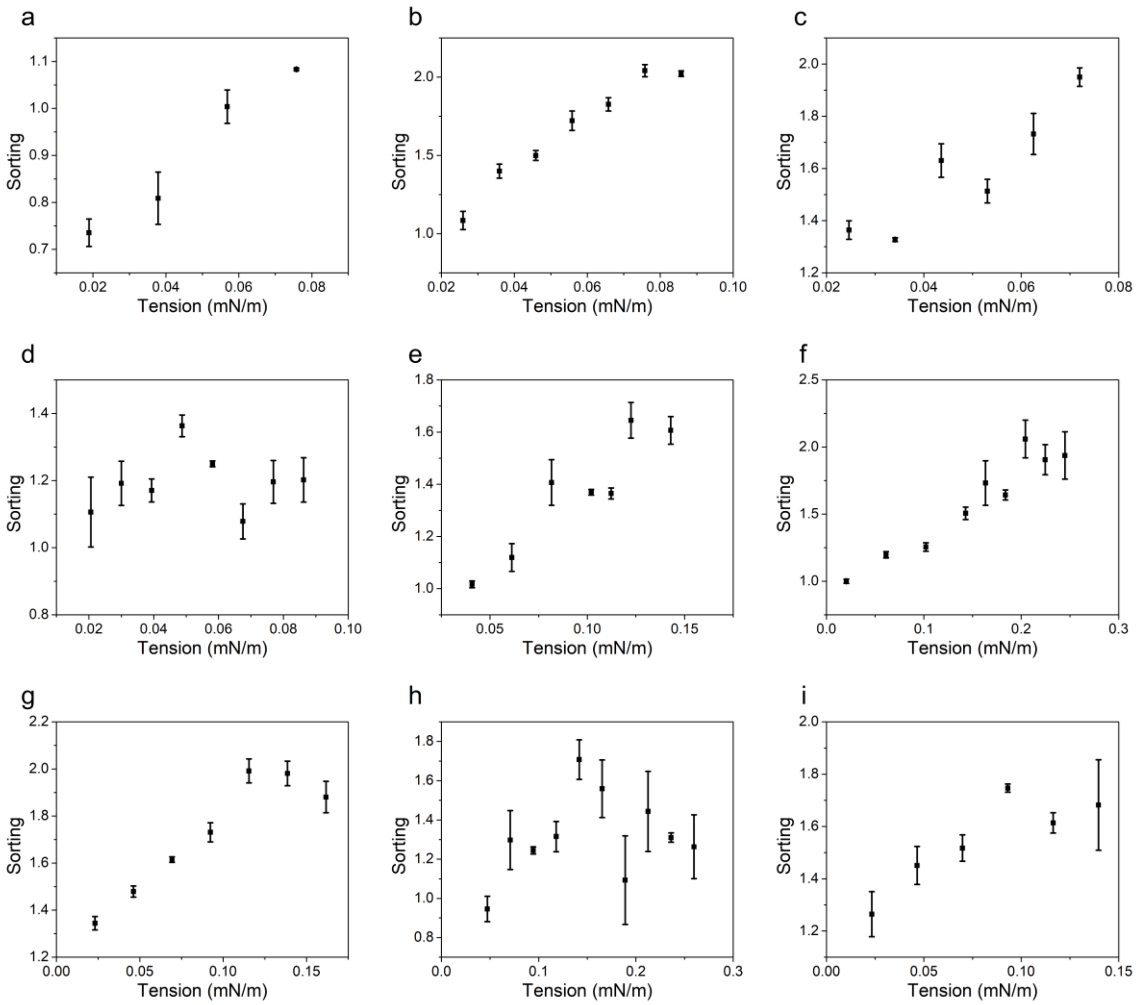

Figure S3. TSPAN4ΔEC1ΔEC2 tube pulling experiment-tension increase. (a-i) Sorting ratio as a function of membrane tension plots of membrane tubes pulled from aspirated GPMVs containing TSPAN4ΔEC1ΔEC2-GFP and dyed with DiI-C12. Each plot represents a different vesicle.

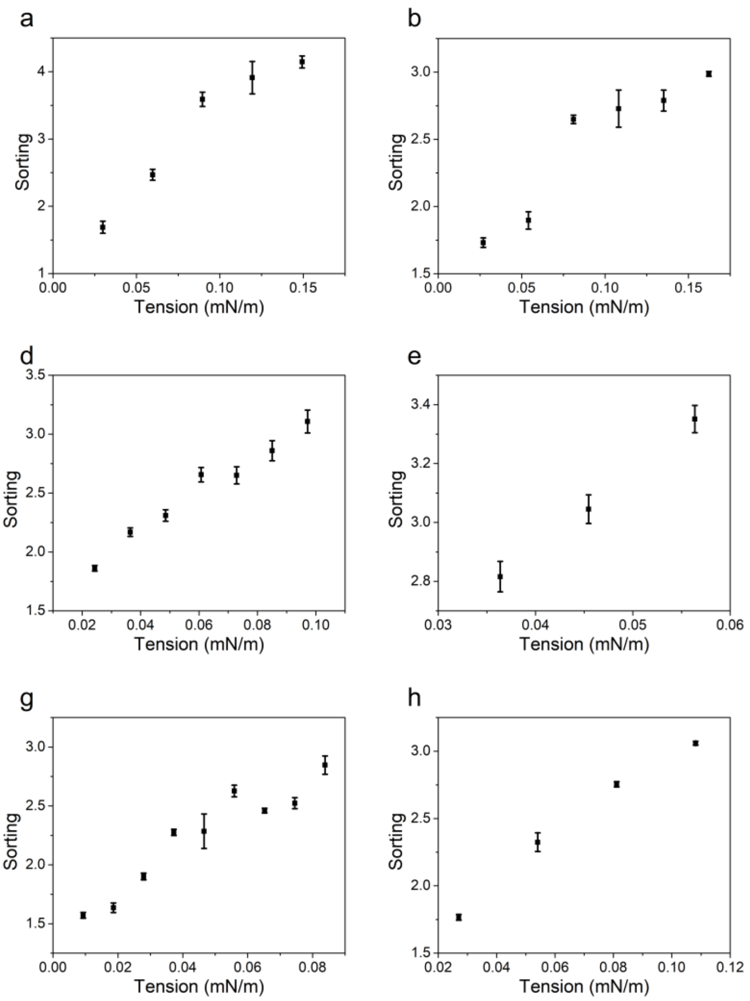

Figure S4. TSPAN4ΔEC1 tube pulling experiment-tension increase. (a-i) Sorting ratio as a function of membrane tension plots of membrane tubes pulled from aspirated GPMVs containing TSPAN4ΔEC1-GFP and dyed with DiI-C12. Each plot represents a different vesicle.

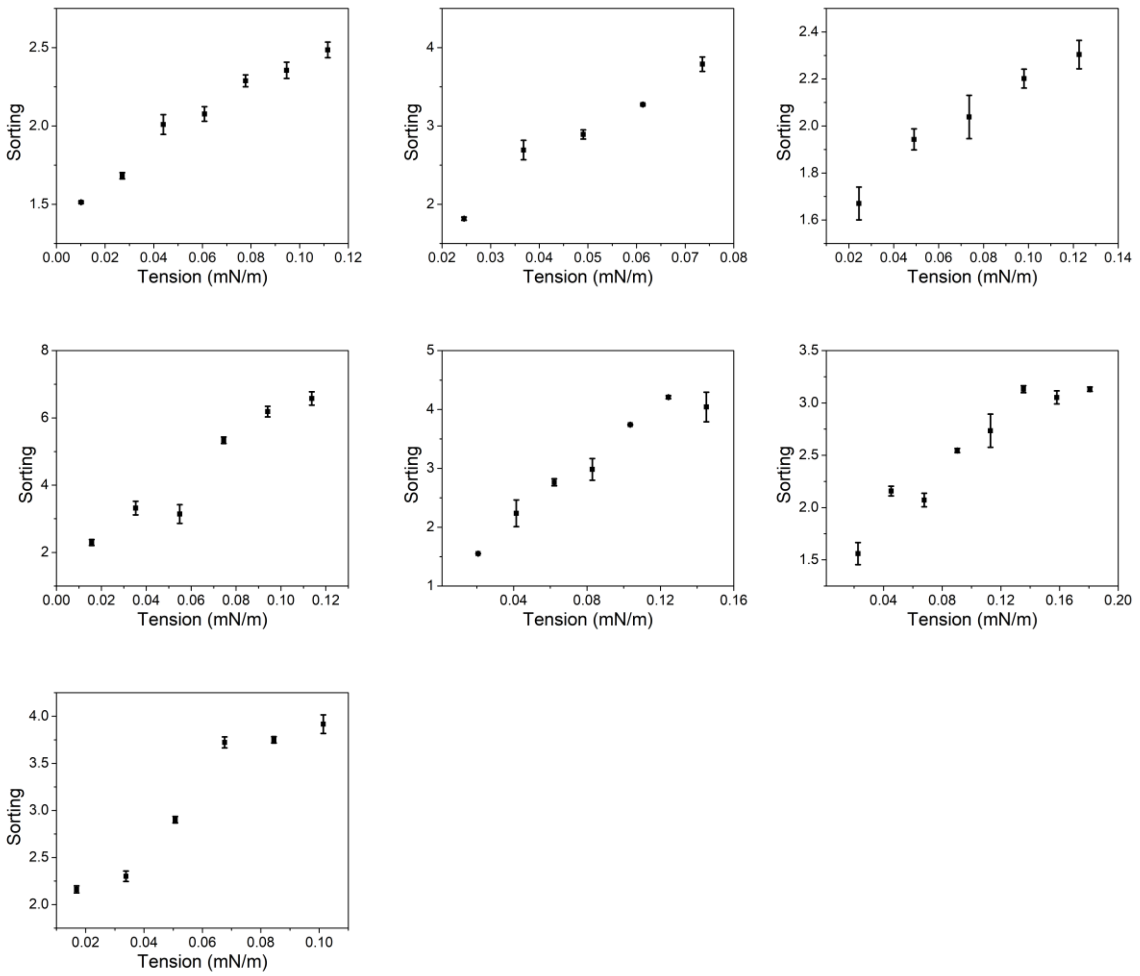

Figure S5. TSPAN4ΔIC tube pulling experiment-tension increase. (a-i) Sorting ratio as a function of membrane tension plots of membrane tubes pulled from aspirated GPMVs containing TSPAN4ΔIC-GFP and dyed with DiI-C12. Each plot represents a different vesicle.

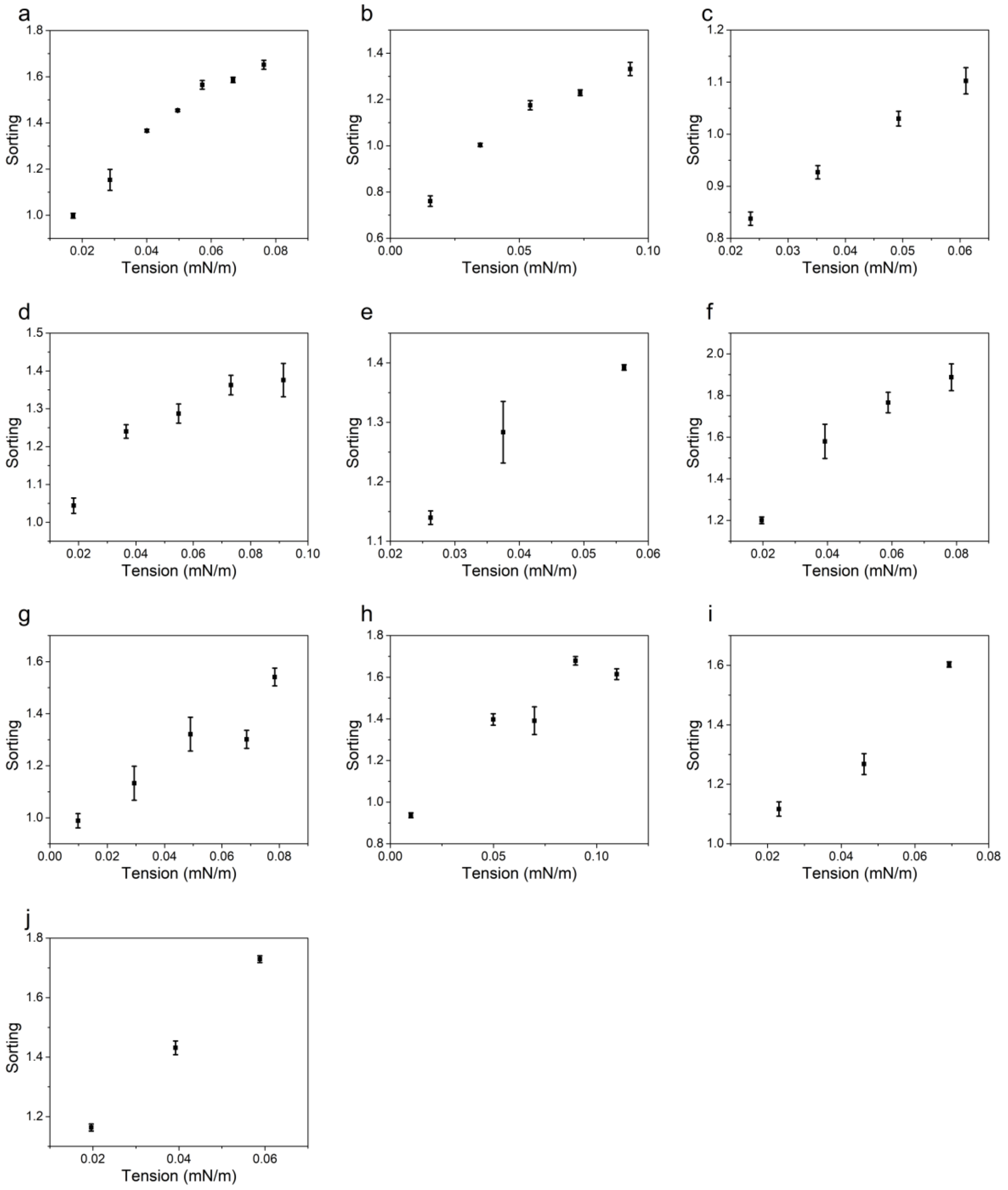

Figure S6. TSPAN4ΔEC2 tube pulling experiment-tension increase. (a-i) Sorting ratio as a function of membrane tension plots of membrane tubes pulled from aspirated GPMVs containing TSPAN4ΔEC2-GFP and dyed with DiI-C12. Each plot represents a different vesicle.

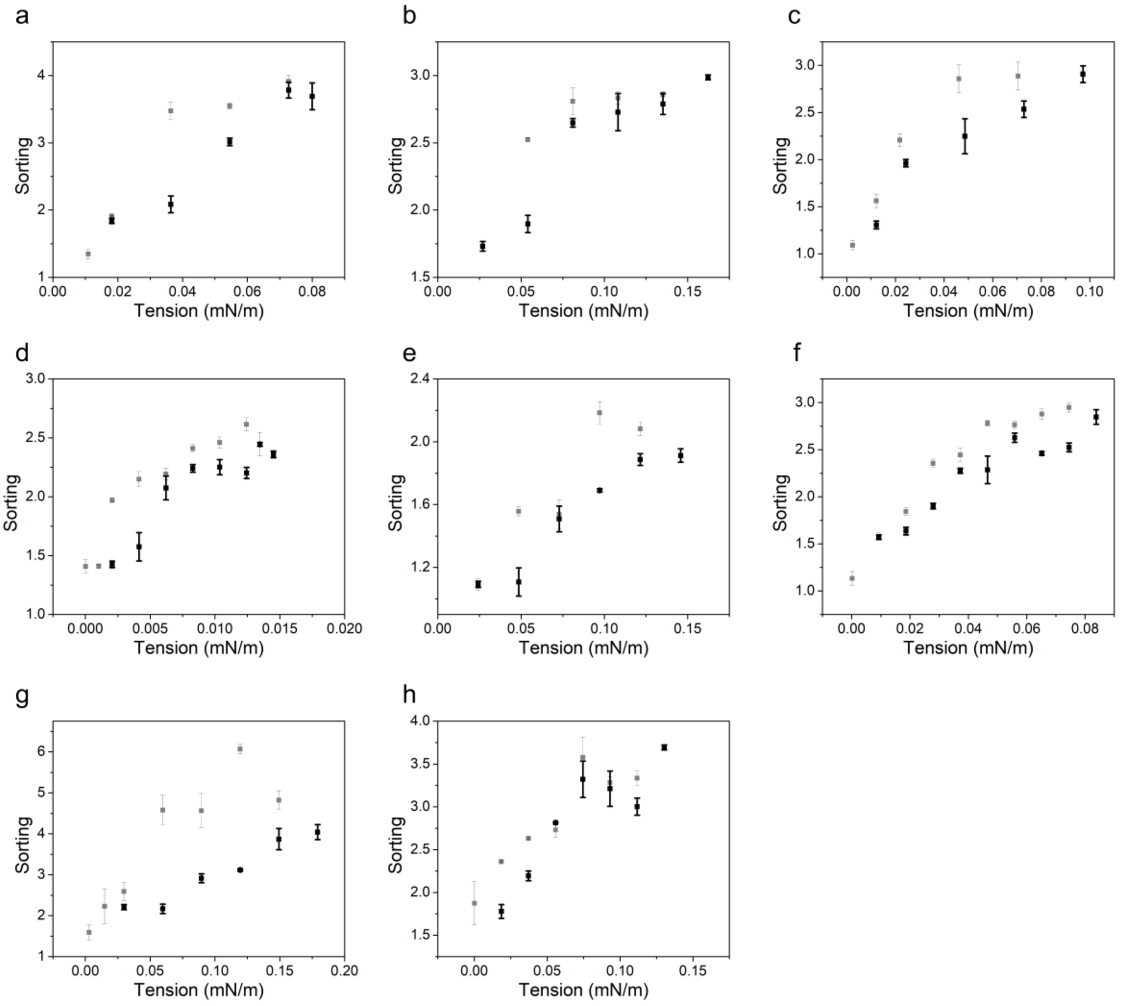

Figure S7. TSPAN4ΔEC1 tube pulling experiment-tension increase followed by tension decrease. (a-i) Sorting ratio as a function of membrane tension plots of membrane tubes pulled from aspirated GPMVs containing TSPAN4ΔEC1-GFP and dyed with DiI-C12. Black and grey squares represent tension increase and tension decrease paths, respectively. Each plot represents a different vesicle.

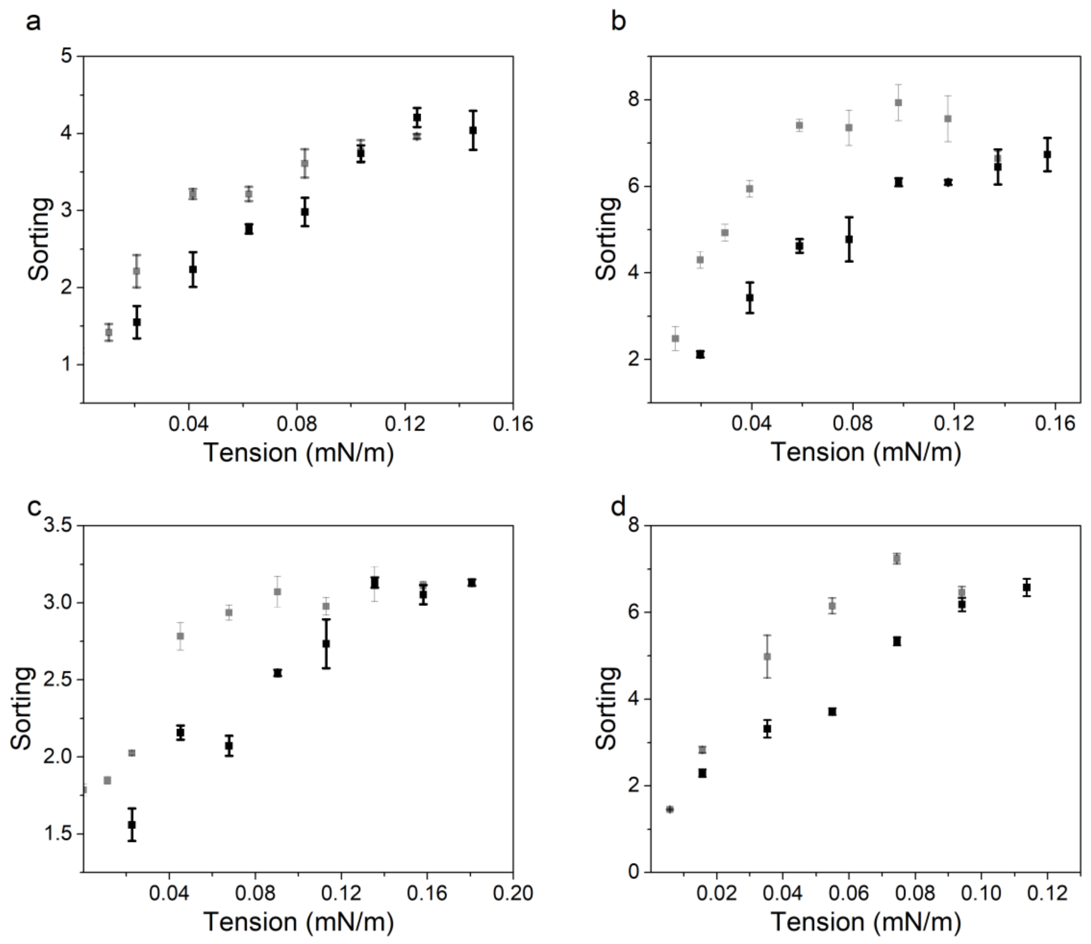

Figure S8. TSPAN4ΔIC tube pulling experiment-tension increase followed by tension decrease. (a-i) Sorting ratio as a function of membrane tension plots of membrane tubes pulled from aspirated GPMVs containing TSPAN4ΔIC-GFP and dyed with DiI-C12. Black and grey squares represent tension increase and tension decrease paths, respectively. Each plot represents a different vesicle.

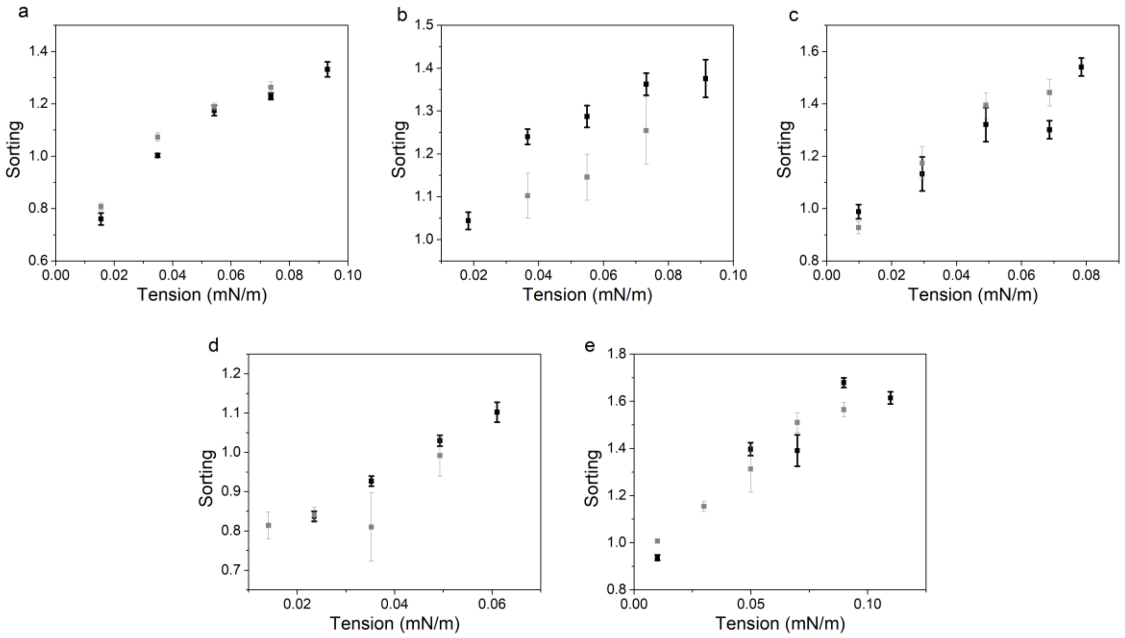

Figure S9. TSPAN4ΔEC2 tube pulling experiment-tension increase followed by tension decrease. (a-i) Sorting ratio as a function of membrane tension plots of membrane tubes pulled from aspirated GPMVs containing TSPAN4ΔEC2-GFP and dyed with DiI-C12. Black and grey squares represent tension increase and tension decrease paths, respectively. Each plot represents a different vesicle.

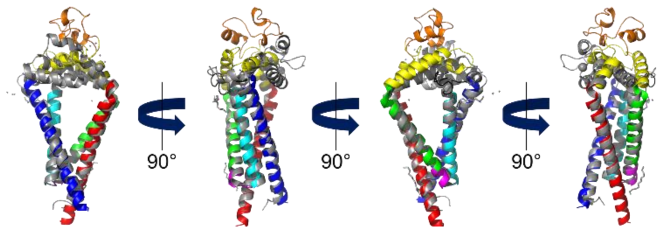

Figure S10. TSPAN4 and CD9 alignment. Alpha fold prediction of TSPAN4 (same color as in Figure1) and crystal structure of CD9 (PDB 6K4J, grey color). The main structural difference between the two proteins is in the EC2 domain (specifically the small loop containing amino acids 151-187 of TSPAN4 which is colored in orange).

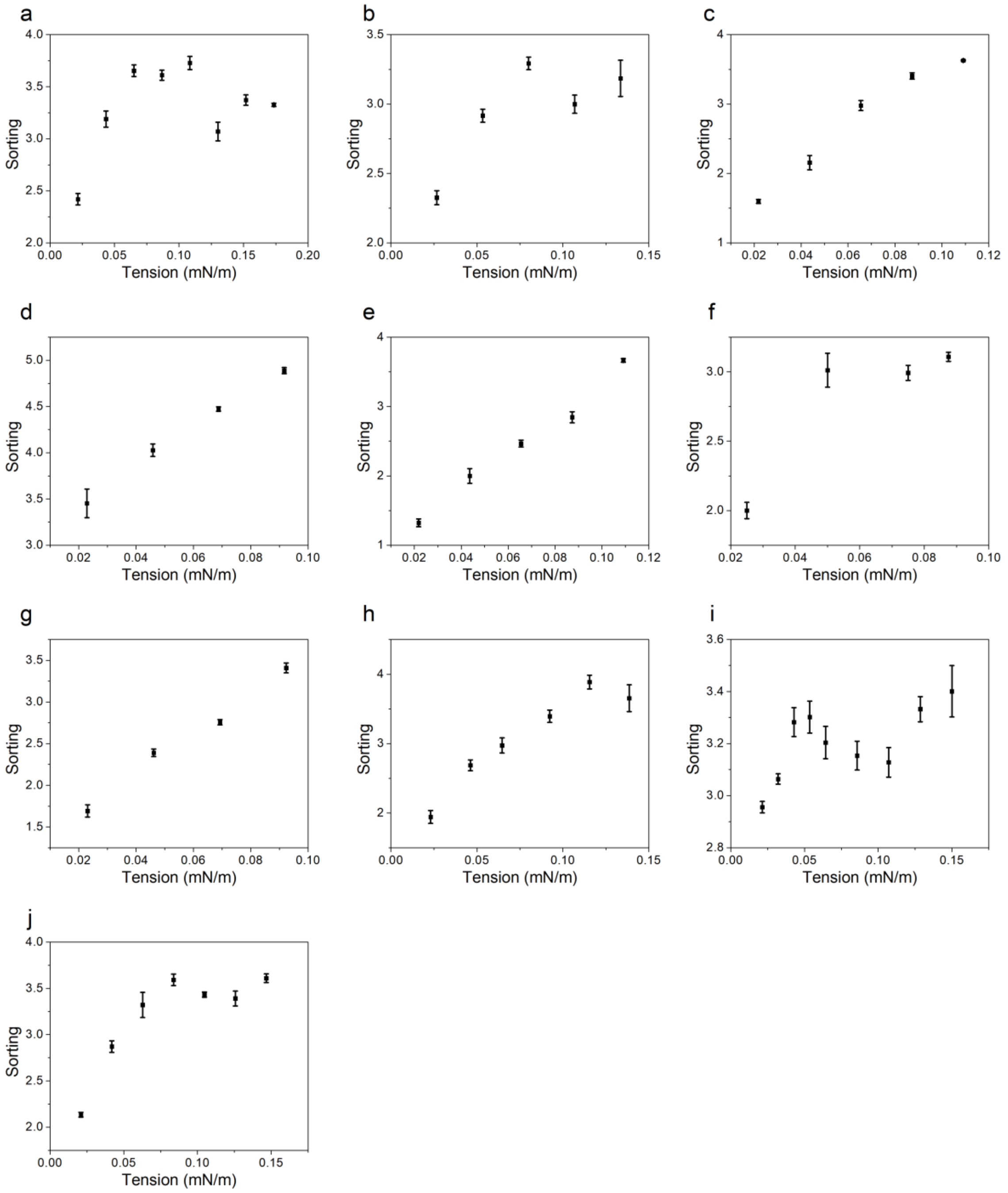

Figure S11. TSPAN4ΔSL tube pulling experiment-tension increase. (a-j) Sorting ratio as a function of membrane tension plots of membrane tubes pulled from aspirated GPMVs containing TSPAN4ΔSL-GFP and dyed with DiI-C12. Each plot represents a different vesicle.

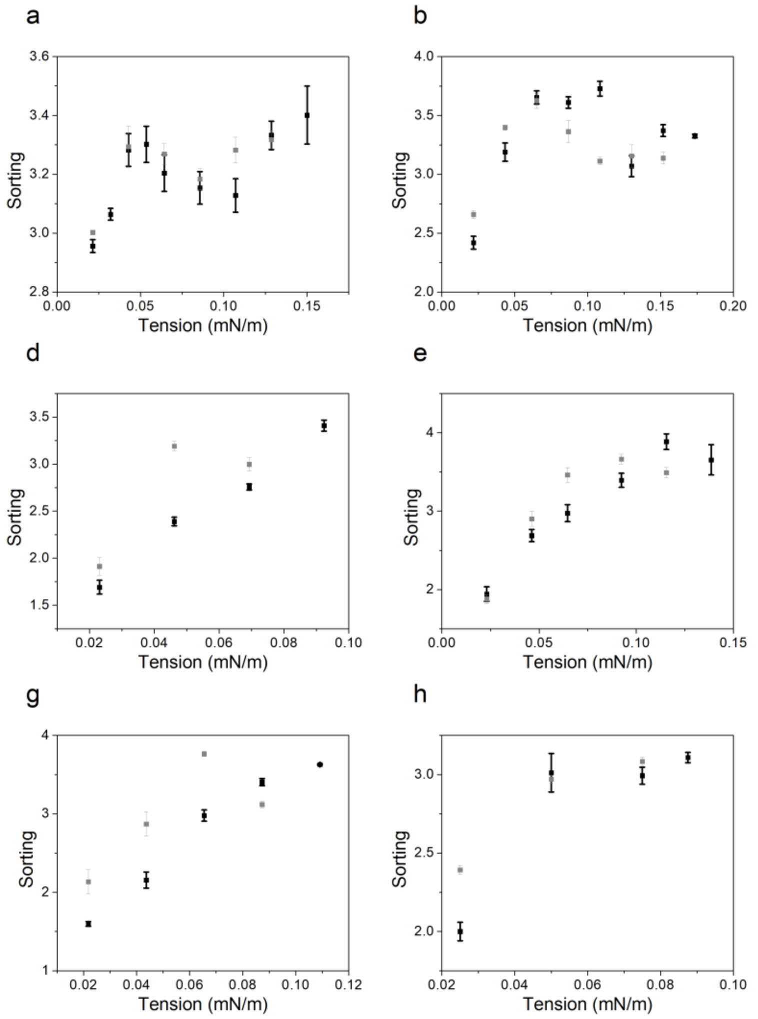

Figure S12. TSPAN4ΔSL tube pulling experiment-tension increase followed by tension decrease. (a-i) Sorting ratio as a function of membrane tension plots of membrane tubes pulled from aspirated GPMVs containing TSPAN4ΔSL-GFP and dyed with DiI-C12. Black and grey squares represent tension increase and tension decrease paths, respectively. Each plot represents a different vesicle.

References:

1. Gerstle, Z., Desai, R. & Veatch, S. L. Giant Plasma Membrane Vesicles: An Experimental Tool for Probing the Effects of Drugs and Other Conditions on Membrane Domain Stability. *Methods Enzymol.* **603**, 129–150 (2018).
