# Supplementary material for: Extracellular domain 2 of TSPAN4 governs its functions
Source: Biophys Rep (N Y). 2024 Mar 5;4(2):100149. doi: 10.1016/j.bpr.2024.100149 (PMC10982557; doi:10.1016/j.bpr.2024.100149)
Supplement: Document S2. Article plus supporting material [file mmc2.pdf]

Biophysical Reports

REPORT

Extracellular domain 2 of TSPAN4 governs its functions

Raviv Dharan,[1,2] Alisa Vaknin,[1,2] and Raya Sorkin[1,2,*]
[1]School of Chemistry, Raymond & Beverly Sackler Faculty of Exact Sciences, Tel Aviv University, Tel Aviv, Israel and [2]Center for Physics and Chemistry of Living Systems, Tel Aviv University, Tel Aviv, Israel

ABSTRACT Tetraspanin 4, a protein with four transmembrane helices and three connecting loops, senses membrane curvature and localizes to membrane tubes. This enrichment in tubular membranes enhances its diverse interactions. While the transmembrane part of the protein likely contributes to curvature sensitivity, the possible roles of the ectodomains in curvature sensitivity of tetraspanin 4 are still unknown. Here, using micropipette aspiration combined with confocal microscopy and optical tweezers, we show that the extracellular loop 2 contributes to the curvature sensitivity and curvature-induced interactions of tetraspanin 4. To this end, we created truncated tetraspanin 4 mutants by deleting each of the connecting loops. Subsequently, we pulled membrane tubes from giant plasma membrane vesicles containing tetraspanin 4-GFP or its mutants while maintaining controllable membrane tension and curvature. Among the mutations tested, the removal of the extracellular loop 2 had the most significant impact on both the curvature sensitivity and interactions of tetraspanin 4. Based on the results, we suggest that the extracellular loop 2 regulates the affinity of tetraspanin 4 towards curved membranes and affects its lateral interactions.

WHY IT MATTERS Tetraspanins are widespread in nearly every cell, showcasing diverse functions linked to crucial cellular and pathological processes like cell adhesion, immune signaling, cell-cell fusion, viral infection, and cancer metastasis. This underscores their significance in cellular mechanisms and suggests potential therapeutic applications. Their various cellular roles are closely tied to their ability to form higher-order structures. The assembly of tetraspanins is likely dependent on their membrane concentration, which increases in curved membranes for some tetraspanins due to their membrane curvature sensitivity. Elucidating the molecular domains governing their curvature sensitivity and interactions is essential for understanding tetraspanin dynamics in the membrane. Here, we demonstrate that the extracellular 2 loop of tetraspanin 4 is crucial for both curvature sensitivity and domain formation, suggesting a way to regulate tetraspanin function.

The tetraspanin family of proteins contains 33 known members in humans that are highly conserved across species (1,2). They participate in numerous cellular processes including adhesion, migration, signaling, fusion, fission, and immune cell functions (3,4,5,6,7). Tetraspanins are known to interact with themselves and other cell surface receptors like integrins, forming tetraspanin-enriched microdomains (8,9,10). One of the tetraspanins that was found to form membrane domains of various sizes is tetraspanin 4 (TSPAN4) (11,12). TSPAN4-enriched domains were found to regulate cellular processes like migrasome formation and cell membrane repair (11,12,13). Like all tetraspanins, TSPAN4 spans the cell membrane with four transmembrane helices (TM1–TM4), which are connected by extracellular and intracellular (IC) loops (Fig. 1 *A*). Extracellular loop 1 (EC1), connecting TM1 and TM2, contains amino acids 39–51. The IC loop strictly connects TM2 and TM3 due to its small length that encompasses amino acids 78–84. EC2, connecting TM3 and TM4, is relatively large and contains amino acids 109–199.

Previously, we demonstrated that TSPAN4 is sensitive to membrane curvature and undergoes significant enrichment in membranes with positive membrane curvature (14). We generated giant plasma membrane vesicles (GPMVs) from HEK293T cells expressing TSPAN4-GFP and labeled them with the plasma membrane dye DiI-C12 (Fig. 1 *B*). We used HEK293T cells since their endogenous TSPAN4 levels are very low compared to TSPAN4-transfected HEK293T cells, as previously demonstrated by mass spectrometry (12). The membrane dye DiI-C12 was chosen after we

Submitted December 26, 2023, and accepted for publication March 1, 2024.

*Correspondence: rsorkin@tauex.tau.ac.il

Editor: Yuval Ebenstein.

https://doi.org/10.1016/j.bpr.2024.100149

© 2024 The Author(s).

This is an open access article under the CC BY license (http://creativecommons.org/licenses/by/4.0/).

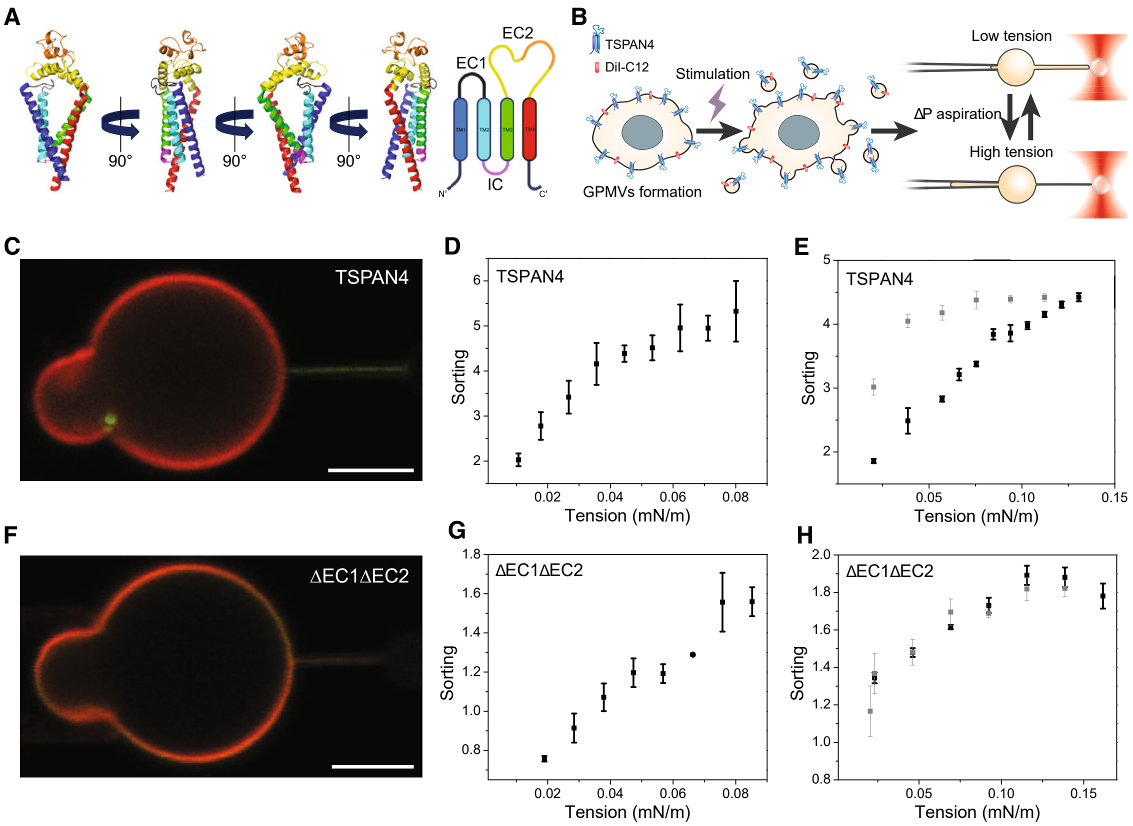

FIGURE 1 Deletion of EC1 and EC2 reduces TSPAN4 sorting in curved membranes. (*A*) Tetraspanin 4 (TSPAN4)-AlphaFold structure prediction illustration. Each structural domain is shown in a different color: TM1, blue; EC1, black; TM2, cyan; IC, magenta; TM3, green; EC2, yellow; and small loop of the EC2, orange. On the right: schematic representation of TSPAN4 structure. (*B*) Illustration of the experimental procedure: formation of giant plasma membrane vesicles (GPMVs) containing TSPAN4 and DiI-C12, followed by a membrane tube-pulling assay. (*C*) Confocal microscopy of a GPMV containing WT TSPAN4-GFP (green) and DiI-C12 (red). (*D*) TSPAN4 sorting as a function of membrane tension plot showing that TSPAN4 sorting increases with membrane tension. (*E*) TSPAN4 sorting as function of membrane tension showing sorting increase with membrane tension followed by sorting hysteresis upon tension decrease. Black squares represent tension increase, whereas gray squares represent tension decrease. (*F*) Confocal microscopy of a GPMV containing TSPAN4ΔEC1ΔEC2-GFP and DiI-C12. (*G* and *H*) TSPAN4ΔEC1ΔEC2 sorting as a function of membrane tension plots. Black squares represent tension increase, whereas gray squares represent tension decrease. All scale bars represent 5 μm. Error bars are SEM. (*C*)–(*E*) are adapted from (14).

examined different membrane dyes for labeling GPMVs (14). By integrating micropipette aspiration with optical tweezers, we pulled membrane tubes from aspirated GPMVs (Fig. 1 *B*). By setting the aspiration pressure in the pipette, we regulated the membrane tension of the vesicle and thus the membrane curvature of the tube (15,16). Using the fluorescence signal of the labeled protein and the lipid dye, we quantified the sorting ratio (17,18):

$$S = \frac{(I_{TSPAN4-GFP}/I_{DiI-C12})_{tube}}{(I_{TSPAN4-GFP}/I_{DiI-C12})_{GPMV}},$$

where I is the fluorescence intensity of GFP or DiI-C12. These measurements revealed that TSPAN4 is progressively sorted into the tube with increasing membrane tension (Fig. 1, *C* and *D*). As TSPAN4 is known to interact with itself and other proteins, the enrichment of TSPAN4 in the tube increased its probability for interactions. To investigate whether TSPAN4 interactions affect the redistribution of TSPAN4, we measured the sorting upon gradual tension increase followed by gradual decrease in the tension (Figs. 1 *B* and S1) and discovered that the sorting exhibited hysteresis (Fig. 1 *E*). Furthermore, we demonstrated that shaving of the extracellular loops of TSPAN4 in the GPMVs resulted in the abolishment of the sorting hysteresis, which suggests that the ECs mediate the curvature-induced TSPAN4 interactions (14).

Here, we set out to uncover the functionality of the ECs of TSPAN4 by implementing the assay described above. We deleted EC1 and EC2 and expressed the new protein TSPAN4ΔEC1ΔEC2-GFP in HEK293T cells. Following GPMV formation, the protein was observed in the membrane of the GPMVs (Fig. S2). Using the tube-pulling assay, we found that the deletion of EC1 and EC2 significantly reduced the curvature sensitivity of TSPAN4 (Fig. 1, *F* and *G*). The

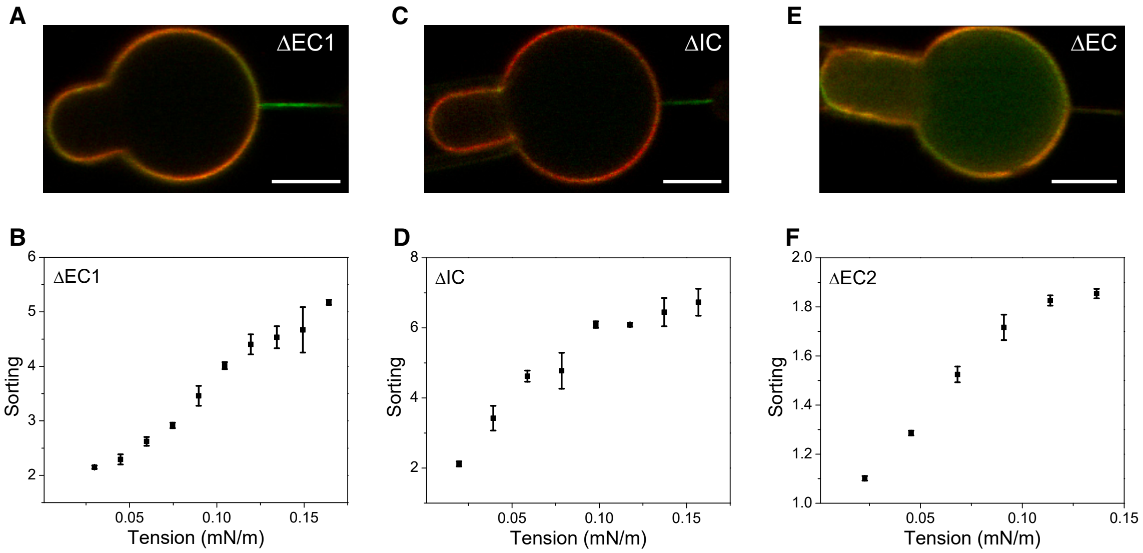

FIGURE 2 Curvature sensitivity of TSPAN4-loop-deletion mutants. (*A–C*) Confocal microscopy images of membrane tubes pulled from aspirated GPMVs dyed with DiI-C12 and containing TSPAN4ΔEC1 (A), TSPAN4ΔIC (B), or TSPAN4ΔEC2 (C). Scale bars represent 5 μm. (*D–F*) Sorting ratio as a function of membrane tension plots of each mutant as indicated. Error bars are SEM.

membrane tube is significantly less enriched with TSPAN4ΔEC1ΔEC2-GPF, as seen from its low green fluorescence intensity (Fig. 1 *F*), compared with the wild-type (WT) TSPAN4 tube fluorescence (Fig. 1 *C*). The sorting values at relatively low membrane tensions, which correspond to tubes with relatively large radii (or low membrane curvature), were approximately one or even lower, indicating that the protein density in the tube was equal to or lower than its density in the flat membrane of the vesicle. When the membrane tension was raised, the sorting of TSPAN4ΔEC1ΔEC2 increased but was still significantly lower compared to the WT-TSPAN4, even at very high membrane curvature (Figs. 1 *G* and S3). Nevertheless, the protein density within tubes exhibiting high membrane curvature was approximately twice as high as that in the flat vesicle, indicating the inherent affinity of TSPAN4 for curved membranes. When the membrane tension was decreased, sorting hysteresis was not observed (Fig. 1 *H*).

When the ECs were shaved in our previous work (14), TSPAN4 curvature sensitivity was not affected. This difference between the results can be explained by the fact that in the WT-TSPAN4 experiments, the shaving of the loops occurred after the protein was embedded in the membrane, whereas in the TSPAN4-ΔEC1ΔEC2, the deletion occurred before the insertion of the protein into the membrane. The protein segments likely to have the greatest impact on the curvature sensitivity of transmembrane proteins are the helices located inside the membrane (19,20,21), i.e., TM1–TM4 for TSPAN proteins. The shaving of the loops following natural insertion into the membrane, with the correct configuration of the transmembrane helices, probably did not significantly alter their orientations, and thereby the sorting was not affected. In

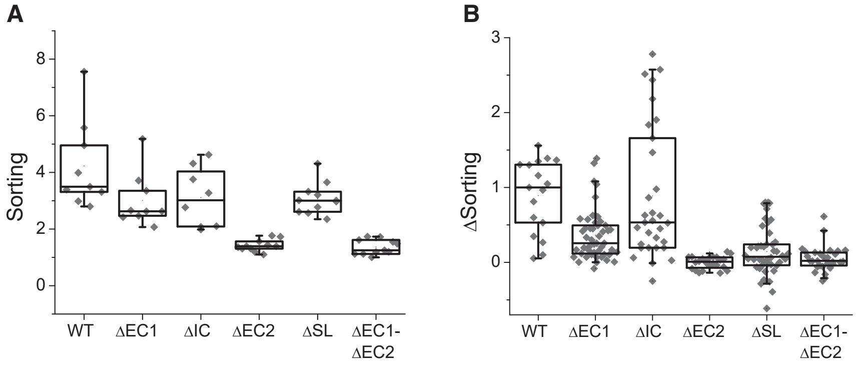

FIGURE 3 Curvature sensitivity and sorting hysteresis of TSPAN4 mutants. (*A*) Boxplot comparing the sorting values of TSPAN4 (WT), TSPAN4ΔEC1, TSPAN4ΔIC, TSPAN4-ΔEC2, TSPAN4ΔSL, and TSPAN4ΔEC1ΔEC2 obtained from tube-pulling experiments conducted at membrane tension of 0.06 ± 0.005 mN/m. From left to right: n = 9, 9, 8, 11, 11, and 10 GPMVs. (*B*) Boxplot comparing the sorting ratio difference between the tension decrease and tension increase paths for the different TSPAN4 mutants. From left to right: n = 3, 9, 5, 6, 10, and 5 GPMVs. Box-whisker plot horizontal lines represent (from bottom to top) the 5%, 25%, 50%, 75%, and 95% of the data.

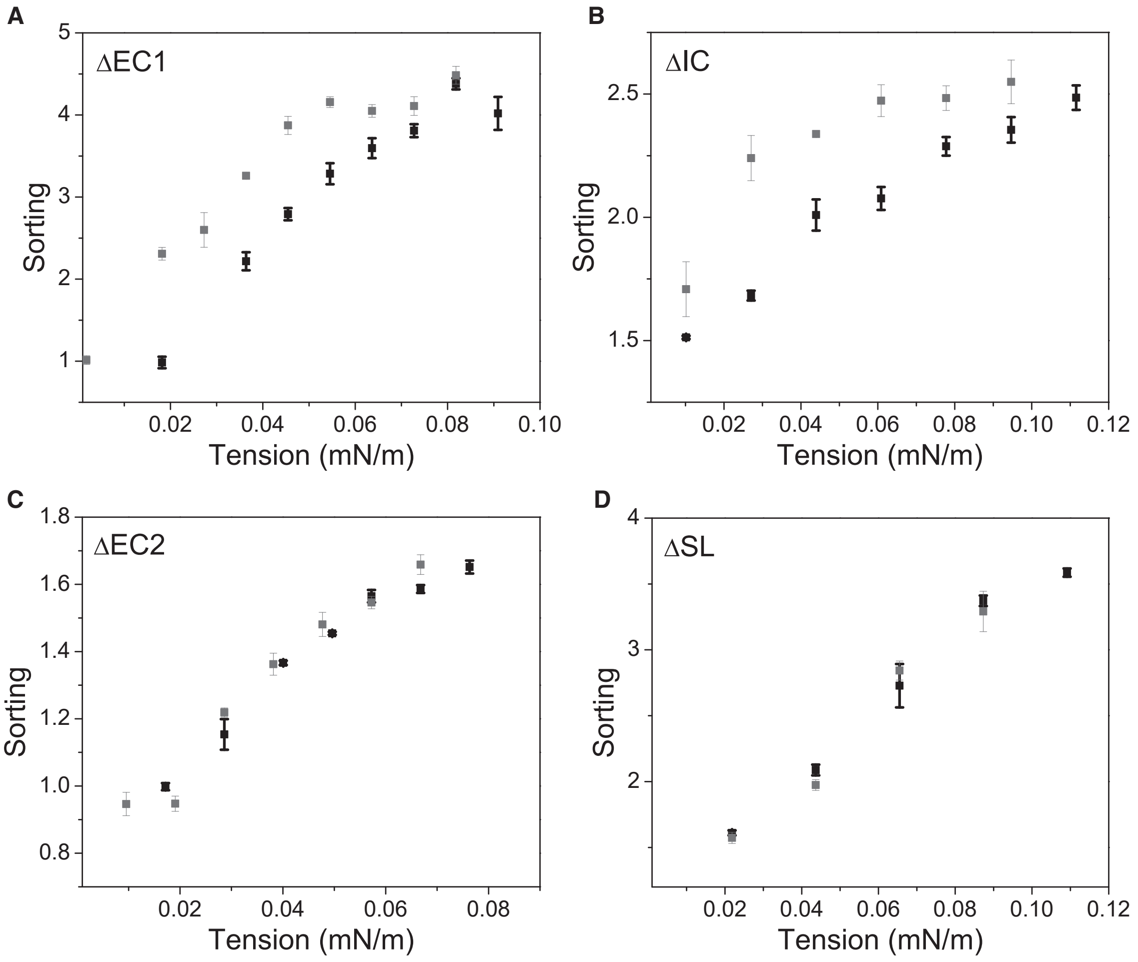

FIGURE 4 EC2 deletion abolished TSPAN4 sorting hysteresis. (*A–D*) Sorting as a function of the membrane tension plots of TPSAN4ΔEC1 (*A*), TSPAN4ΔIC (*B*), TSPAN4ΔEC2 (*C*), and TSPAN4ΔSL (*D*). In all images, black squares represent tension increase, whereas gray squares represent tension decrease. Error bars are SEM.

the TSPAN4ΔEC1ΔEC2 mutant, the loops that connected TM1–TM2 and TM3–TM4 were absent during the protein synthesis, and thereby the orientations of these TMs were probably altered compared to the WT-TSPAN4.

In order to examine the impact of each loop connecting the four transmembrane helices on TSPAN4 sorting, we deleted each loop separately. First, we deleted the EC1 from the TSPAN4 and repeated the tube-pulling experiment. The deletion did not significantly affect the sorting of TSPAN4 into the membrane tubes (Fig. 2 *A*). Sorting was evident even at relatively low membrane tension and substantially increased with further tension increase (Figs. 2 *B* and S4). Next, we deleted the IC loop, which connects the second and third transmembrane helices of TSPAN4. Similarly to the EC1 deletion results, the deletion of IC loop did not affect the tubular enrichment of TSPAN4 (Fig. 3 *C*), and the sorting ratio reached high values (Figs. 2 *D* and S5). We next deleted the EC2 from the protein. This deletion drastically affected TSPAN4 membrane distribution and reduced its tubular enrichment (Fig. 2 *E*). Similarly to the TSPAN4ΔEC1ΔEC2 results, the sorting values at low tension were close to one (Figs. 2 *F* and S6) and increased to around two at high membrane tension. Fig. 3 *A* shows the sorting values of the different TSPAN4 mutants obtained from experiments conducted under the same membrane tension. The results show that the deletion of EC2, compared to the deletion of EC1 and IC, significantly reduced the sorting of TSPAN4 into curved membranes. The reduction in the curvature sensitivity for TSPAN4ΔEC2 implies that the EC2 contributes to the conical structure of TSPAN4. The most variable region among different TSPANs is the EC2 domain, and it is associated with the diverse interactions observed among various TSPANs (22,23). Our results suggest that the EC2 may also be crucial for the curvature sensitivity of TSPANs.

Next, we wanted to examine the sorting hysteresis of TSAPN4 with the truncated mutants that we created. The sorting values for increasing tension were evaluated for each of the mutants and compared to sorting values observed upon tension decrease (Fig. 3 *B*). Like the curvature sensitivity results, TSPAN4ΔEC1 and TSPAN4ΔIC showed hysteresis in the sorting values, whereas TSPAN4ΔEC2 did not (Figs. 3 *B* and 4, *A–C*). The sorting hysteresis of TSPAN4ΔEC1 was lower compared to WT-TSPAN4 (Figs. 3 *B*, 4 *A*, and S7), implying that the interactions were likely impaired for this mutant. Deletion of IC did not significantly affect the sorting hysteresis of TSPAN4 (Figs. 3 *B*, 4 *B*, and S8). The sorting hysteresis of TSPAN4ΔEC2, however, was completely abolished (Figs. 3 *B*, 4 *C*, and S9), suggesting that EC2 is the dominant part that mediates TSAPN4 curvature-induced interactions. The reduced interactions of TSPAN4ΔEC2 can result from the reduced protein enrichment in the tube and not only from the absence of EC2.

To further explore how EC2 contributes to TSPAN4 curvature-induced interactions, we created a new mutant with deletion of a small loop (SL) from the EC2 domain. The SL, containing amino acids 151–187, is one of the main structural differences between CD9 and TSPAN4 according to the CD9 crystal structure and AlphaFold prediction of TSPAN4 (24,25,26). The alignment of the proteins shows that all other regions, including the transmembrane helices, have almost identical configurations (Fig. S10) and thereby probably the same curvature affinity. Our previous results (14) showed that, indeed, the proteins have the same intrinsic curvature; however, they differ in the sorting hysteresis, suggesting that the SL contributes to TSPAN4 interactions. TSPAN4ΔSL was sensitive to membrane curvature and exhibited high enrichment in the tube (Figs. 3 *B*, 4 *D*, and S11). The sorting hysteresis, however, was significantly reduced (Figs. 3 B, 4 *D*, and S12). The fact that the SL deletion affects the sorting hysteresis and not the curvature sensitivity suggests that the absence of the EC2 in TSPAN4ΔEC2 was the leading reason for the lack of curvature-induced interactions rather than the reduced tube enrichment. It is important to note that the interactions mediated by the EC2, leading to the sorting hysteresis, could involve TSPAN4 self-interactions, interactions with other TSPANs, and/or interactions with other types of proteins (8,27,28).

Overall, we investigated each of the loops that connect the four TMs of TSPAN4. Our results show that the EC1 and IC loops do not influence the curvature sensitivity of TSPAN4. Furthermore, the IC loop does not affect TSPAN4 curvature-induced interactions, whereas EC1 seems to be involved in interactions to some extent. Notably, the EC2 was crucial for TSPAN4 curvature-induced interactions. Likewise, it contributes to the curvature sensitivity of the protein probably by allowing the correct conical configuration of the TMs. Although the curvature sensitivity was significantly damaged in TSPAN4ΔEC2 and TSPAN4ΔEC1EΔC2, the protein was still enriched in the highly curved membrane, likely due to its intrinsic conical structure.

TSPAN proteins are crucial for essential cellular processes including fertilization and migrasome formation (12,29). TSPAN4 was found to become enriched in curved membranes and to associate into membrane domains which are crucial for cellular processes like migrasome formation (11). The EC2 protein ectodomain includes a cysteine-cysteine-glycine motif and cysteine residues, which are thought to be essential for efficient intercellular interactions between TSPANs and other associated proteins (30,31). As TSPANs' mode of action is highly linked to their molecular interactions and membrane domain formation, the EC2 domain might be a regulator of TSPAN proteins that determines their level of enrichment in curved membranes and governs their interactions with other molecules. Hence, it is plausible that the EC2 domain serves as crucial region for modulating the biological functions of TSPANs.

SUPPORTING MATERIAL

Supplemental information can be found online at https://doi.org/10.1016/j.bpr.2024.100149.

AUTHOR CONTRIBUTIONS

R.D., A.V., and R.S. designated the research; R.D. and A.V. performed the experiments; R.D. analyzed data; and R.D. and R.S. wrote the manuscript.

ACKNOWLEDGMENTS

R.S. acknowledges the support by the Israel Science Foundation (grant no. 1289/20) and the NSF-BSF (grant no. 2021793) and holds the Raymond and Beverly Sackler Career Development Chair for Young Faculty. This work was co-funded by the European Union (ERC ReMembrane 101077502). The views and opinions expressed are, however, those of the authors only and do not necessarily reflect those of the European Union or the European Research Council Executive Agency. Neither the European Union nor the granting authority can be held responsible for them.

DECLARATION OF INTERESTS

The authors declare no competing interests.

DECLARATION OF GENERATIVE AI AND AI-ASSISTED TECHNOLOGIES IN THE WRITING PROCESS

During the preparation of this work, the authors used Chat GPT 3.5 in order to improve the grammatical structure of several sentences. After using this tool/service, the authors reviewed and edited the content as needed and take full responsibility for the content of the publication.

REFERENCES

1. Le Naour, F., E. Rubinstein, …, C. Boucheix. 2000. Severely reduced female fertility in CD9-deficient mice. *Science.* 287:319–321.
2. Charrin, S., F. le Naour, …, E. Rubinstein. 2009. Lateral organization of membrane proteins : tetraspanins spin their web. *Biochem. J.* 420:133–154.
3. Hemler, M. E. 2003. Tetraspanin Proteins Mediate Cellular Penetration, Invasion, and Fusion Events and Define a Novel Type of Membrane Microdomain. *Annu. Rev. Cell Dev. Biol.* 19:397–422.
4. Boucheix, C., and E. Rubinstein. 2001. *Cell. Mol. Life Sci.* 58:1189–1205.
5. Jankovičová, J., P. Sečová, …, J. T. Antalíková. 2020. more than markers of extracellular vesicles in reproduction. *Int. J. Mol. Sci.* 21:1–30.
6. Levy, S., S. C. Todd, and H. T. Maecker. 1998. CD81 (TAPA-1): A molecule involved in signal transduction and cell adhesion in the immune system. *Annu. Rev. Immunol.* 16:89–109.
7. Tarrant, J. M., L. Robb, …, M. D. Wright. 2003. Tetraspanins: Molecular organisers of the leukocyte surface. *Trends Immunol.* 24:610–617.
8. Rubinstein, E. 2011. The complexity of tetraspanins. *Biochem. Soc. Trans.* 39:501–505.
9. Le Naour, F., M. André, …, E. Rubinstein. 2006. Membrane microdomains and proteomics: Lessons from tetraspanin microdomains and comparison with lipid rafts. *Proteomics.* 6:6447–6454.
10. Hemler, M. E. 2005. Tetraspanin functions and associated microdomains. *Nat. Rev. Mol. Cell Biol.* 6:801–811.
11. Huang, Y., B. Zucker, …, L. Yu. 2019. Migrasome formation is mediated by assembly of micron-scale tetraspanin macrodomains. *Nat. Cell Biol.* 21:991–1002.
12. Dharan, R., Y. Huang, …, R. Sorkin. 2023. Tetraspanin 4 stabilizes membrane swellings and facilitates their maturation into migrasomes. *Nat. Commun.* 14, 1037.
13. Huang, Y., X. Zhang, …, L. Yu. 2022. Assembly of Tetraspanin-enriched macrodomains contains membrane damage to facilitate repair. *Nat. Cell Biol.* 24:825–832.
14. Dharan, R., S. Goren, R. Sorkin…, 2022. Transmembrane protein tetraspinin 4 and CD9 sense membrane curvature. *PNAS.* https://doi.org/10.1101/2022.06.02.494291.
15. Hochmuth, R. M., and E. A. Evans. 1982. Extensional flow of erythrocyte membrane from cell body to elastic tether. I. Analysis. *Biophys. J.* 39:71–81.
16. Sorre, B., A. Callan-Jones, …, A. Roux. 2012. Nature of curvature coupling of amphiphysin with membranes depends on its bound density. *Proc. Natl. Acad. Sci. USA.* 109:173–178.
17. Beltrán-Heredia, E., F. C. Tsai, …, F. Monroy. 2019. Membrane curvature induces cardiolipin sorting. *Commun. Biol.* 2:225.
18. Moreno-Pescador, G., C. D. Florentsen, …, P. M. Bendix. 2019. Curvature- A nd Phase-Induced Protein Sorting Quantified in Transfected Cell-Derived Giant Vesicles. *ACS Nano.* 13:6689–6701.
19. Kluge, C., M. Pöhnl, and R. A. Böckmann. 2022. Spontaneous local membrane curvature induced by transmembrane proteins. *Biophys. J.* 121:671–683.
20. Strahl, H., S. Ronneau, …, L. W. Hamoen. 2015. Transmembrane protein sorting driven by membrane curvature. *Nat. Commun.* 6, 8728.
21. McMahon, H. T., and E. Boucrot. 2015. Membrane curvature at a glance. *J. Cell Sci.* 128:1065–1070.
22. Seigneuret, M., A. Delaguillaumie, …, H. Conjeaud. 2001. Structure of the tetraspanin main extracellular domain: A partially conserved fold with a structurally variable domain insertion. *J. Biol. Chem.* 276:40055–40064.
23. Susa, K. J., A. C. Kruse, and S. C. Blacklow. 2023. Tetraspanins: structure, dynamics, and principles of partner-protein recognition. *Trends Cell Biol.* 1–14. https://doi.org/10.1016/j.tcb.2023.09.003.
24. Umeda, R., Y. Satouh, …, O. Nureki. 2020. Structural insights into tetraspanin CD9 function. *Nat. Commun.* 11:1606.
25. Jumper, J., R. Evans, …, D. Hassabis. 2021. Highly accurate protein structure prediction with AlphaFold. *Nature.* 596:583–589.
26. Varadi, M., S. Anyango, …, S. Velankar. 2022. AlphaFold Protein Structure Database: Massively expanding the structural coverage of protein-sequence space with high-accuracy models. *Nucleic Acids Res.* 50:D439–D444.
27. Schmidt, S. C., A. Massenberg, …, T. Lang. 2024. Microscopic clusters feature the composition of biochemical tetraspanin-assemblies and constitute building-blocks of tetraspanin enriched domains. *Sci. Rep.* 14:2093.
28. Charrin, S., S. Manié, …, E. Rubinstein. 2003. Multiple levels of interactions within the tetraspanin web. *Biochem. Biophys. Res. Commun.* 304:107–112.
29. Runge, K. E., J. E. Evans, …, D. G. Myles. 2007. Oocyte CD9 is enriched on the microvillar membrane and required for normal microvillar shape and distribution. *Dev. Biol.* 304:317–325.
30. Levy, S., and T. Shoham. 2005. The tetraspanin web modulates immune-signalling complexes. *Nat. Rev. Immunol.* 5:136–148.
31. Hopf, T. A., L. J. Colwell, …, D. S. Marks. 2012. Theory Three-Dimensional Structures of Membrane Proteins from Genomic Sequencing. *Cell.* 149:1607–1621.

BPR, Volume *4*

Supplemental information

Extracellular domain 2 of TSPAN4 governs its functions

Raviv Dharan, Alisa Vaknin, and Raya Sorkin

Supplementary material for

Extracellular domain 2 of TSPAN4 governs its functions

Raviv Dharan[1,2], Alisa Vaknin[1,2] and Raya Sorkin*[1,2]

1. School of Chemistry, Raymond & Beverly Sackler Faculty of Exact Sciences, Tel Aviv University, Israel
2. Center for Physics and Chemistry of Living Systems, Tel Aviv University, Tel Aviv, Israel

*Corresponding author: Raya Sorkin, Email: rsorkin@tauex.tau.ac.il

Materials and methods

TSPAN4 mutants expression plasmid preparation.

The complementary DNA of mouse-TSPAN4 was sub-cloned into the pEGFP-N1 vector (Addgene). To generate the following truncated versions of TSPAN4 (UniProt accession number Q9DCK3): deletion of the amino acids 39-51 (TSPAN4ΔEC1), 78-84 (TSPAN4ΔIC), 109-199 (TSPAN4ΔEC2), 39-51 and 109-199 (TSPAN4ΔEC1ΔEC2), or 151-187 (TSPAN4ΔSL), we applied the reverse PCR approach. First, a PCR was performed, generating a linear vector using the following primers:

Construct name	Primers (5' → 3')
TSPAN4ΔEC1	Forward: TCGGCTGCCAACCTGCTCA<br>Reverse: CTGTGTGGCAGCCAACCAGATGC
TSPAN4ΔEC2	Forward: GAGAACCTGCTAGCTGTGGGCATCTTTGGA<br>Reverse: ACTGTAGGCAAAGAAGAGCACAGCAATGG
TSPAN4ΔEC1ΔEC2	Same as for TSPAN4ΔEC1 and TSPAN4ΔEC2 template
TSPAN4ΔIC	Forward: ACTTTCTTTGTGCTGCTGCTGCTAGTGTT<br>Reverse: CTTGAGGGCCCCAATGCAGCCCA
TSPAN4ΔSL	Forward: TCGCCCTGTTATGAGACAGTGAAGGCC<br>Reverse: AACTCCACAGCATCGGAAATCAGTCTGGAT

The product was applied to DpnI (NEB, R0176) residual methylated DNA digestion, followed by purification using the NucleoSpin Gel and PCR-Clean-up (Macherey-Nagel 740609). Next, the linear vector was phosphorylated using the T4 polynucleotide kinase (NEB, M0201) at 37 °C for 30 minutes and followed by a deactivation step for 20 minutes at 65 °C. Subsequently, the phosphorylated product was used for a ligation reaction with T4 DNA ligase (NEB, M0202) for 2 hours at room temperature. Finally, 5 µL of the ligated product was transformed into chemically competent DH5α *Escherichia coli* cells. A colony was inoculated, followed by

plasmid extraction (Macherey-Nagel, 740727), and sent to Sanger sequencing (ZABAM Instrumentation and Service sequencing unit at Tel Aviv University).

Cell transfection and GPMV formation

HEK293T (ATCC CRL-3216TM) cells were cultured at 37°C and 5% CO_2 in DMEM (Gibco-Thermo Fisher scientific 11995065) supplemented with 10% Fetal bovine serum (biological industries, 04-001-1A) and 1% penicillin-streptomycin (Gibco-Thermo Fisher scientific 15140122). 24 hours before cell transfection, the cells were plated in DMEM supplemented with 10% Fetal bovine serum at 20% confluency in a 25 cm^2 flask (Romical) coated with poly-L-lysine (Sigma-Aldrich P6282) to keep the cells attached during the blebbing process and to minimize cell debris in solution. At 50% confluency, cells were transiently transfected with 5 µg DNA using Lipofectamine 2000 (Invitrogen, Thermo Fisher scientific) according to the manufacture's protocols and then grown 24 hours for protein expression. GPMVs were produced according to a published protocol[1]. Briefly, following TSPAN4-GFP (or the different TSPAN4 mutants) expression the cell membrane was stained with DiI-C12 membrane dye (Invitrogen, Thermo Fisher scientific D383), washed with GPMV buffer (10 mM HEPES, 150 mM NaCl, 2 mM CaCl2, pH 7.4) twice, and incubated with 1 mL of GPMV buffer containing 1.9 mM DTT (Sigma-Aldrich 1019777701) and 27.6 mM formaldehyde (Sigma-Aldrich F8775). Secreted GPMVs were then collected and isolated from the cells and immediately used for optical trapping experiments. In order to verify the presence of TSPAN4-GFP in the GPMV membrane, we scanned the GPMVs using 488 laser before each measurement.

Tube pulling from aspirated GPMVs

The experiments were performed using a C-trap® confocal fluorescence optical tweezers setup (LUMICKS, Amsterdam, the Netherlands) made of an inverted microscope based on a water-immersion objective (NA 1.2) together with a condenser top lens placed above the sample. The optical traps are generated by splitting a 10W 1064-nm laser into two orthogonally polarized, independently steerable optical traps. To steer the two traps, one coarse-positioning piezo stepper mirror and one accurate piezo mirror were used. Optical traps were used to capture polystyrene microbeads. The displacement of the trapped beads from the center of the trap was measured and converted into a force signal by back-focal plane interferometry of the condenser lens using two position-sensitive detectors. The samples were illuminated by a bright field 850-nm LED and imaged in

transmission onto a metal-oxide semiconductor (CMOS) camera. **Confocal fluorescence microscopy:** The C-Trap uses a 3 color, fiber-coupled laser with wavelengths 488, 561 and 638 nm for fluorescence excitation. Scanning was done using a fast tip/tilt piezo mirror. For confocal detection, the emitted fluorescence was descanned, separated from the excitation by a dichroic mirror, and filtered using an emission filters (Blue: 500-550 nm, Green: 575-625 nm and Red: 650-750 nm). Photons were counted using fiber-coupled single-photon counting modules. The multimode fibers serve as pinholes providing background rejection. For confocal imaging the 488 nm and 532 nm lasers were used for GFP and DiI-C12 excitation with 5% and 1% laser power respectively, 54.34 µW is the maximal laser power, and the emission detected in three channels (Blue, Green, Red).

Experimental chamber: PDMS walls were placed on the bottom cover slip (Thorlabs CG15KH1) and mounted onto an automated XY-stage. The GPMVs were added to the chamber and after about 15 minutes, a few drops of oil were put on the sample surface to prevent evaporation. A micropipette aspiration setup including micromanipulator (Sensapex) holding a micropipette with diameter of 5 µm (BioMedical instruments) connected to a Fluigent EZ-25 pump was integrated to our optical tweezers instrument. Before each experiment, the zero-suction pressure was found by aspirating a 3.43 µm polystyrene bead (Spherotech) into the pipette and reducing the suction pressure until the bead stopped moving. A membrane tube was pulled from aspirated GPMVs using beads trapped by the optical tweezers. First, a membrane tube was pulled at relatively low suction pressure (0.05-0.1 mbar). Then we gradually increased the suction pressure (usually by 0.05-0.1 mbar steps) until we reached values in the range of 0.3-0.7 mbar. In the experiments where the membrane tube was not ruptured during the tension increase, we gradually decreased the pressure stepwise, until reaching the zero pressure.

Data Analysis

Data acquisition was carried out using Bluelake, a commercial software from Lumicks. This software stores experimental data acquired with the C-trap in HDF5 files, which can be processed using Lumicks' Pylake python package. Images of the confocal scans were reconstituted from photon count per pixel data in the HDF5 files using Pylake. All data analysis was performed with custom-written Python scripts. Fluorescence intensity profiles were obtained from the images by averaging the photon count of the relevant fluorescent channel (Blue or Green) in the region of interest.

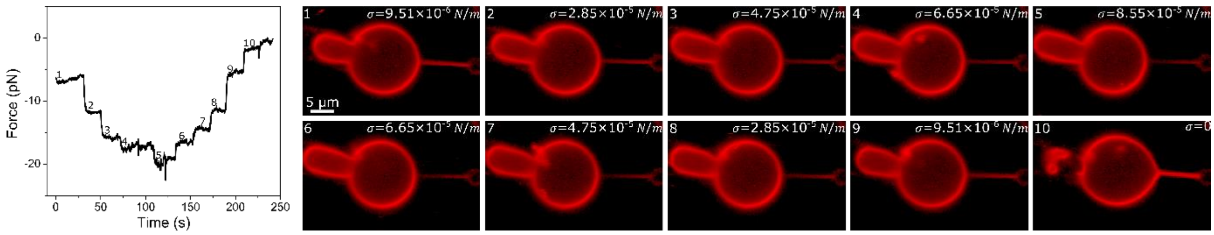

Figure S1. Tube pulling experiment- membrane tension increase followed by tension decrease. On the left, a plot showing the force to hold a tube as function of time during tension increase steps followed by subsequent tension decrease of aspirated GPMVs labelled with DiI-C12. The numbers indicate to the corresponding confocal images on the right presenting the values of the membrane tension applied by the micropipette aspiration. The image pixel intensities are presented in logarithmic scale. It can be seen that when the membrane tension was increased (1-5) the pulling force and the length of the tongue of the vesicle inside the pipette increased as well, whereas the radius of the tube decreased. When the membrane tension was decreased (6-10), the trends were reversed.

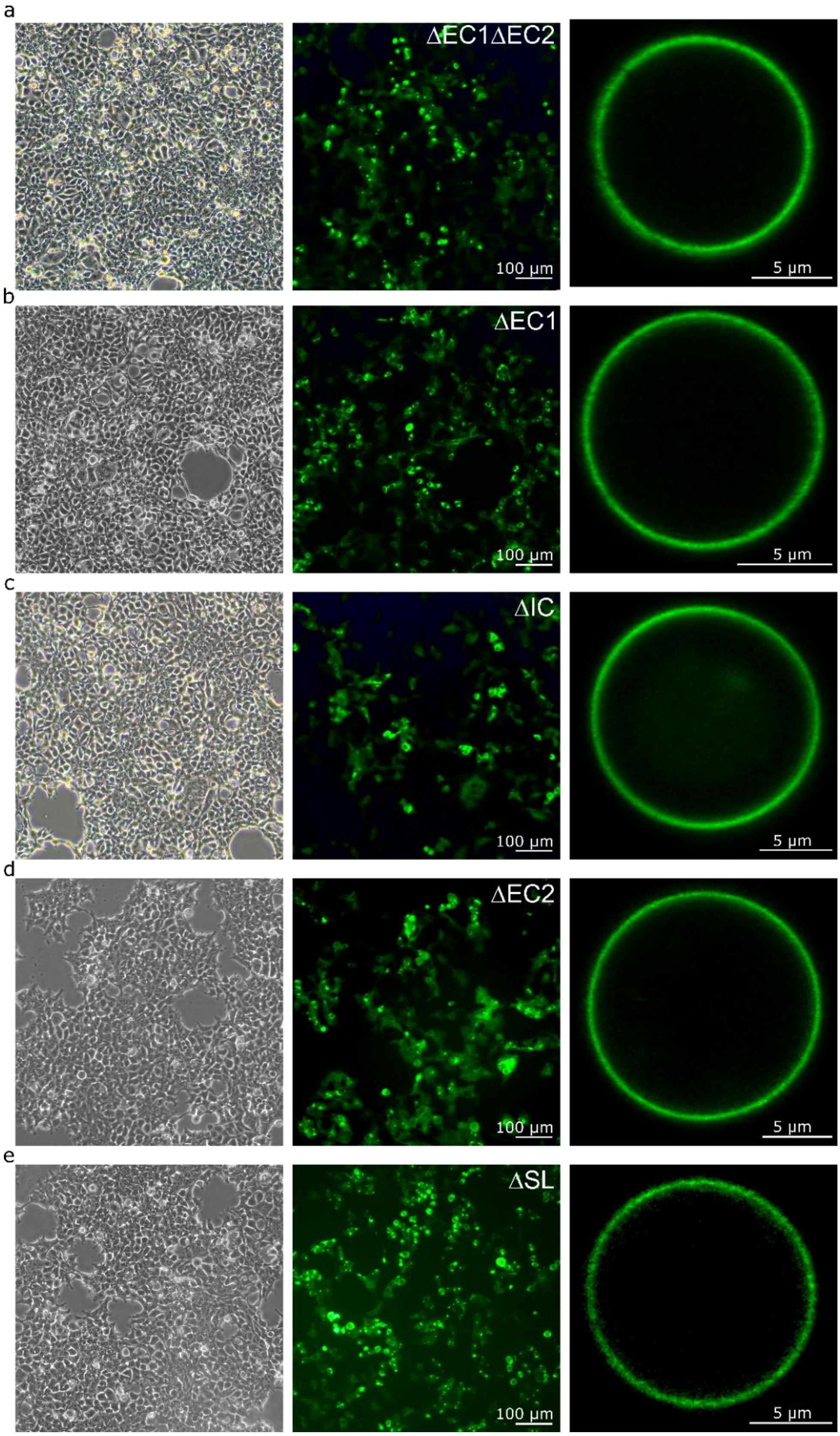

Figure S2. TSPAN4 mutants' expression. (a-e) Bright field (left) and green channel (middle) microscopy images of HEK293T cells expressing TSPAN4ΔEC1ΔEC2-GFP (a), TSPAN4ΔEC1-GFP (b), TSPAN4ΔIC-GFP (c), TSPAN4ΔEC2-GFP (d) and TSPAN4ΔSL-GFP (e). On the right, confocal microscopy images of isolated GPMVs containing TSPAN4 mutants in their membrane.

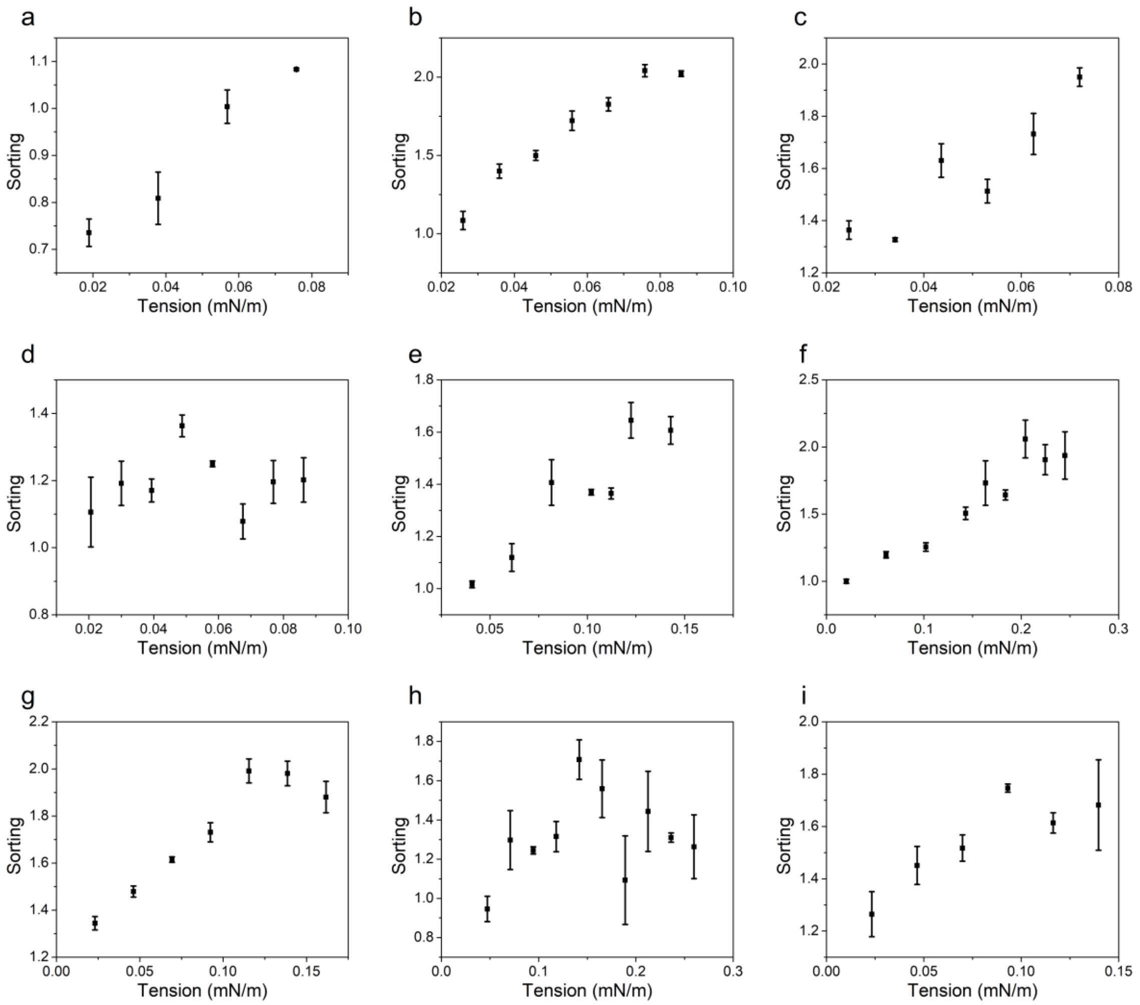

Figure S3. TSPAN4ΔEC1ΔEC2 tube pulling experiment-tension increase. (a-i) Sorting ratio as a function of membrane tension plots of membrane tubes pulled from aspirated GPMVs containing TSPAN4ΔEC1ΔEC2-GFP and dyed with DiI-C12. Each plot represents a different vesicle.

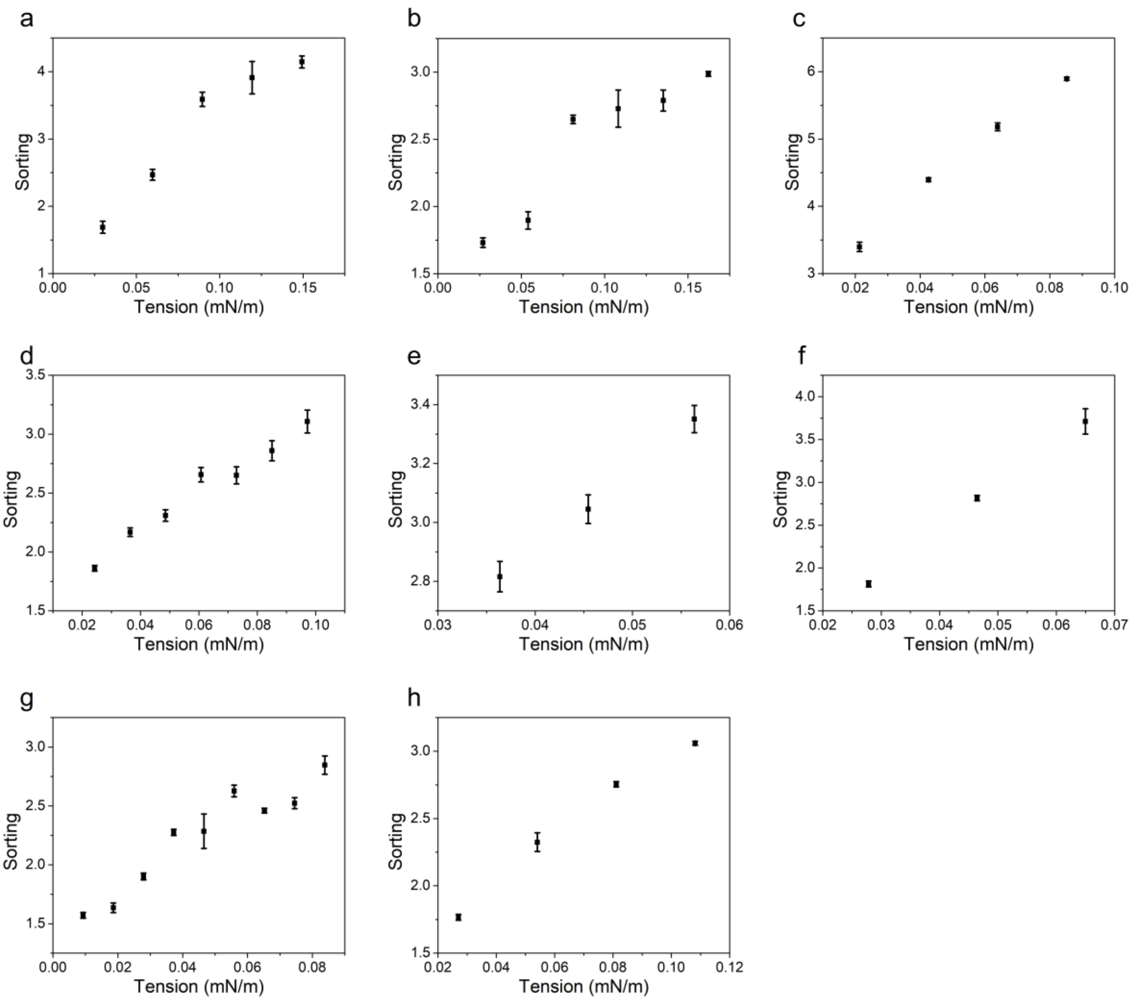

Figure S4. TSPAN4ΔEC1 tube pulling experiment-tension increase. (a-i) Sorting ratio as a function of membrane tension plots of membrane tubes pulled from aspirated GPMVs containing TSPAN4ΔEC1-GFP and dyed with DiI-C12. Each plot represents a different vesicle.

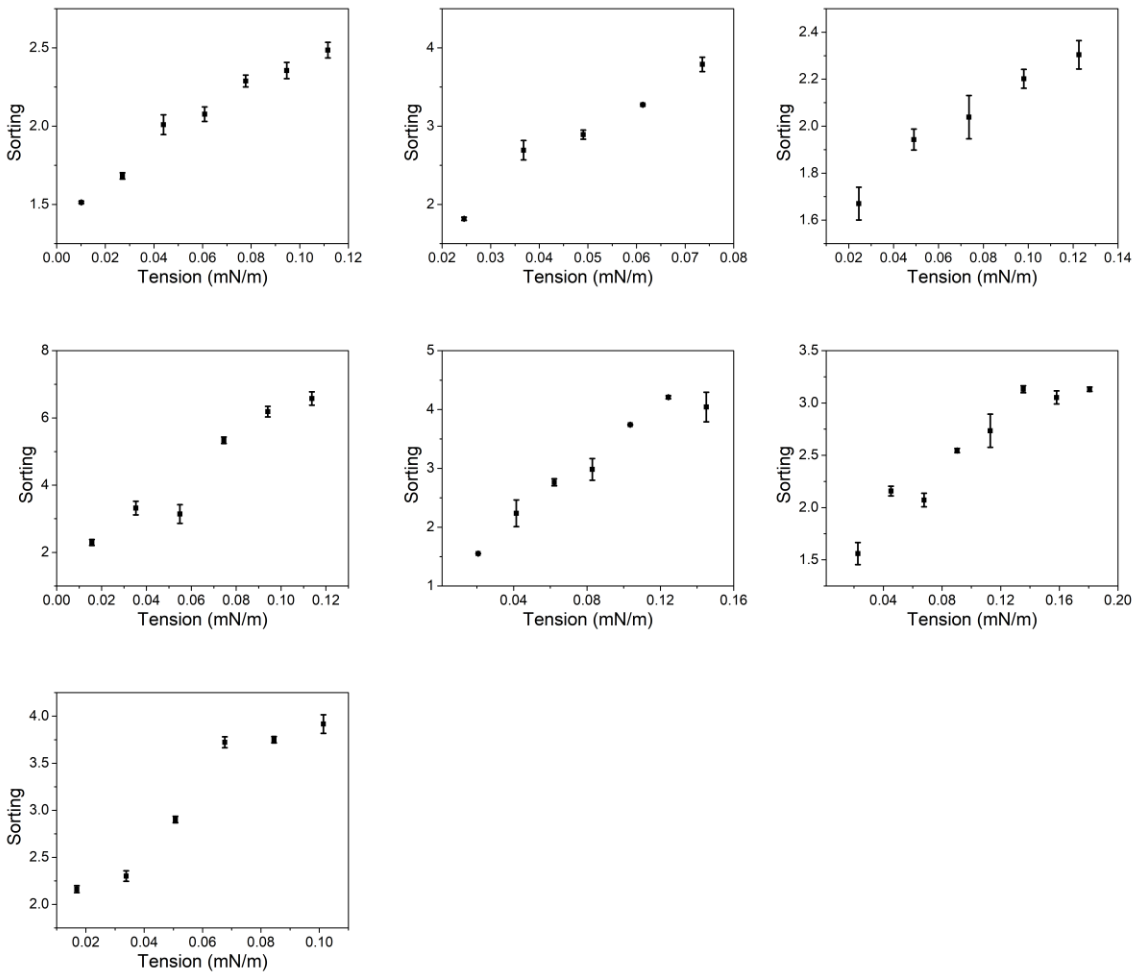

Figure S5. TSPAN4ΔIC tube pulling experiment-tension increase. (a-i) Sorting ratio as a function of membrane tension plots of membrane tubes pulled from aspirated GPMVs containing TSPAN4ΔIC-GFP and dyed with DiI-C12. Each plot represents a different vesicle.

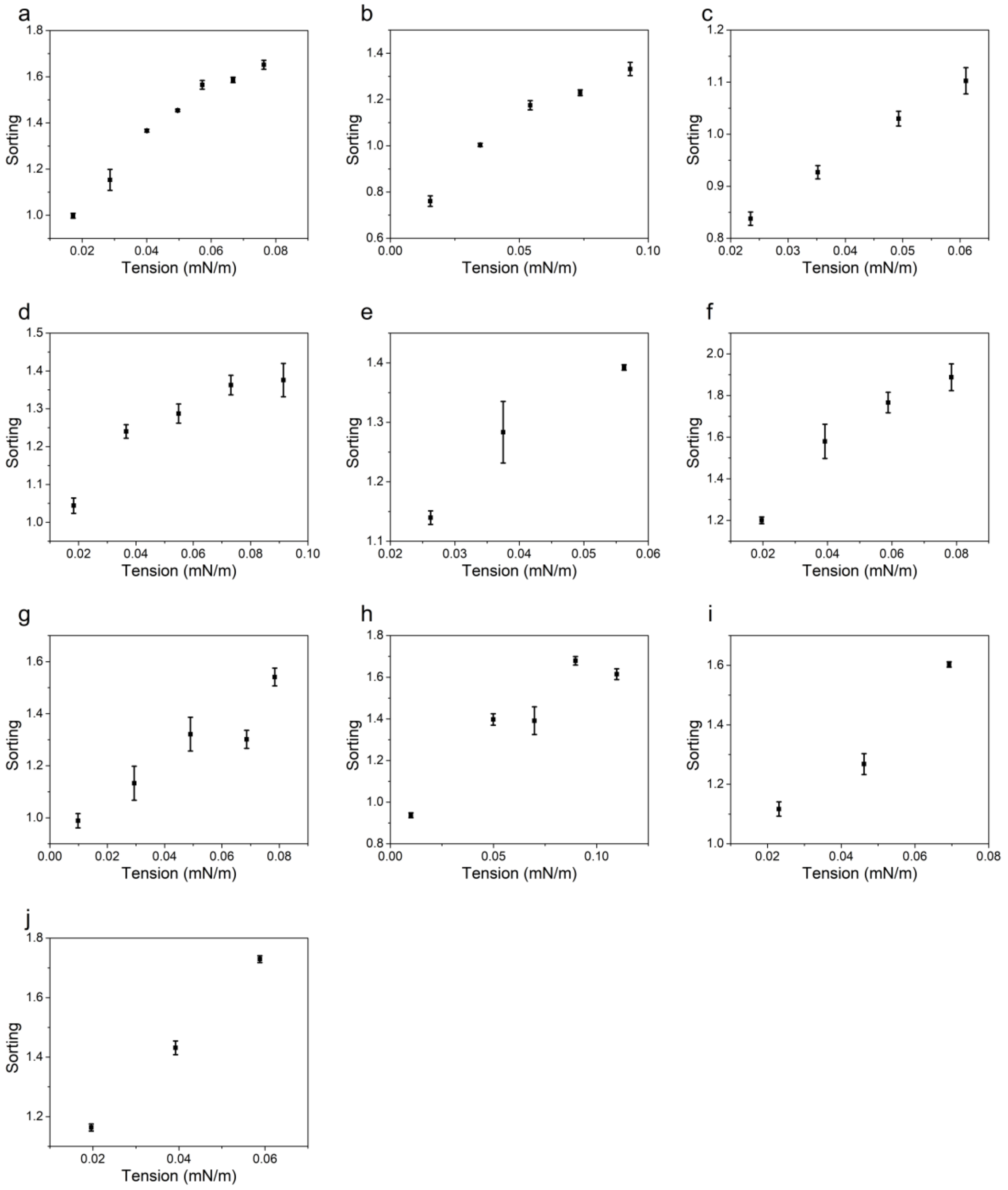

Figure S6. TSPAN4ΔEC2 tube pulling experiment-tension increase. (a-i) Sorting ratio as a function of membrane tension plots of membrane tubes pulled from aspirated GPMVs containing TSPAN4ΔEC2-GFP and dyed with DiI-C12. Each plot represents a different vesicle.

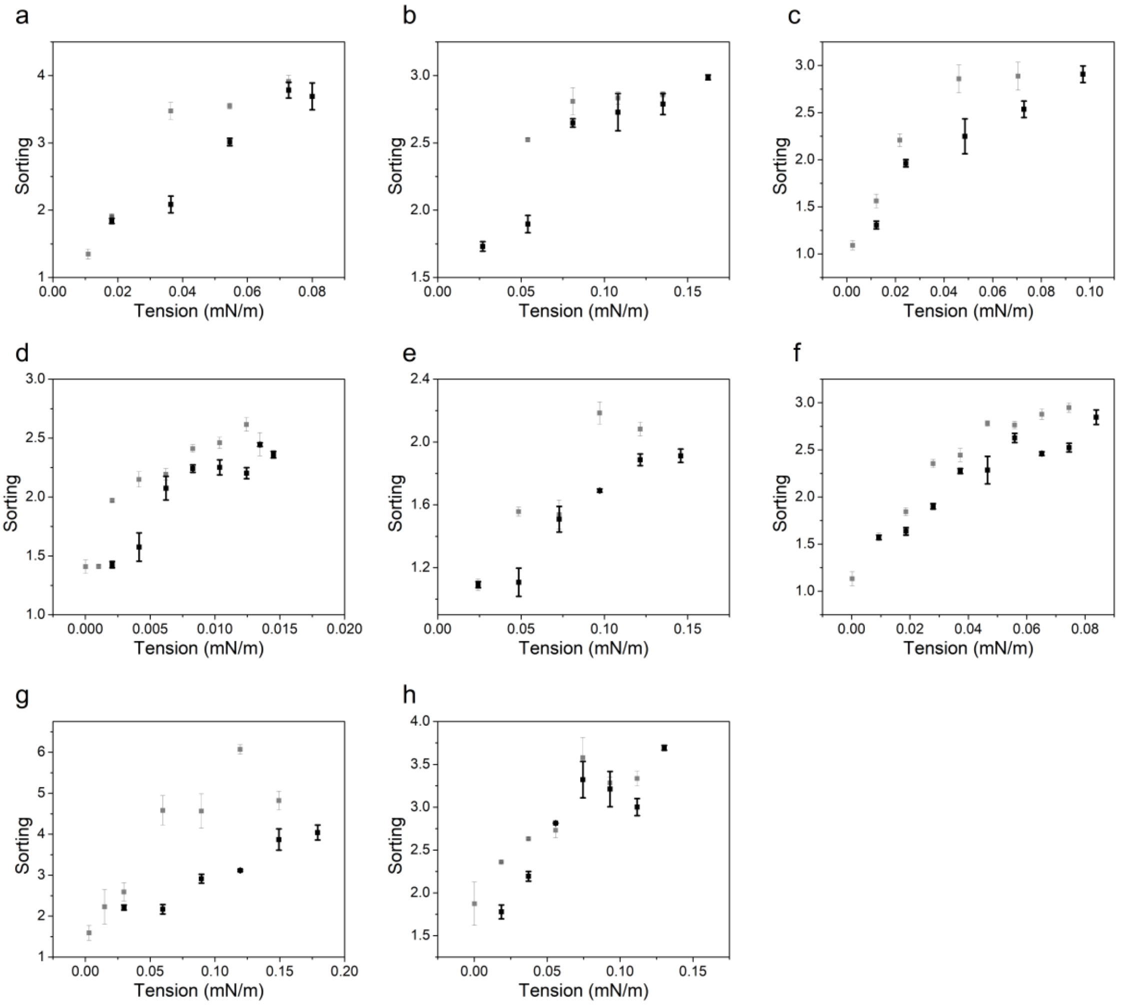

Figure S7. TSPAN4ΔEC1 tube pulling experiment-tension increase followed by tension decrease. (a-i) Sorting ratio as a function of membrane tension plots of membrane tubes pulled from aspirated GPMVs containing TSPAN4ΔEC1-GFP and dyed with DiI-C12. Black and grey squares represent tension increase and tension decrease paths, respectively. Each plot represents a different vesicle.

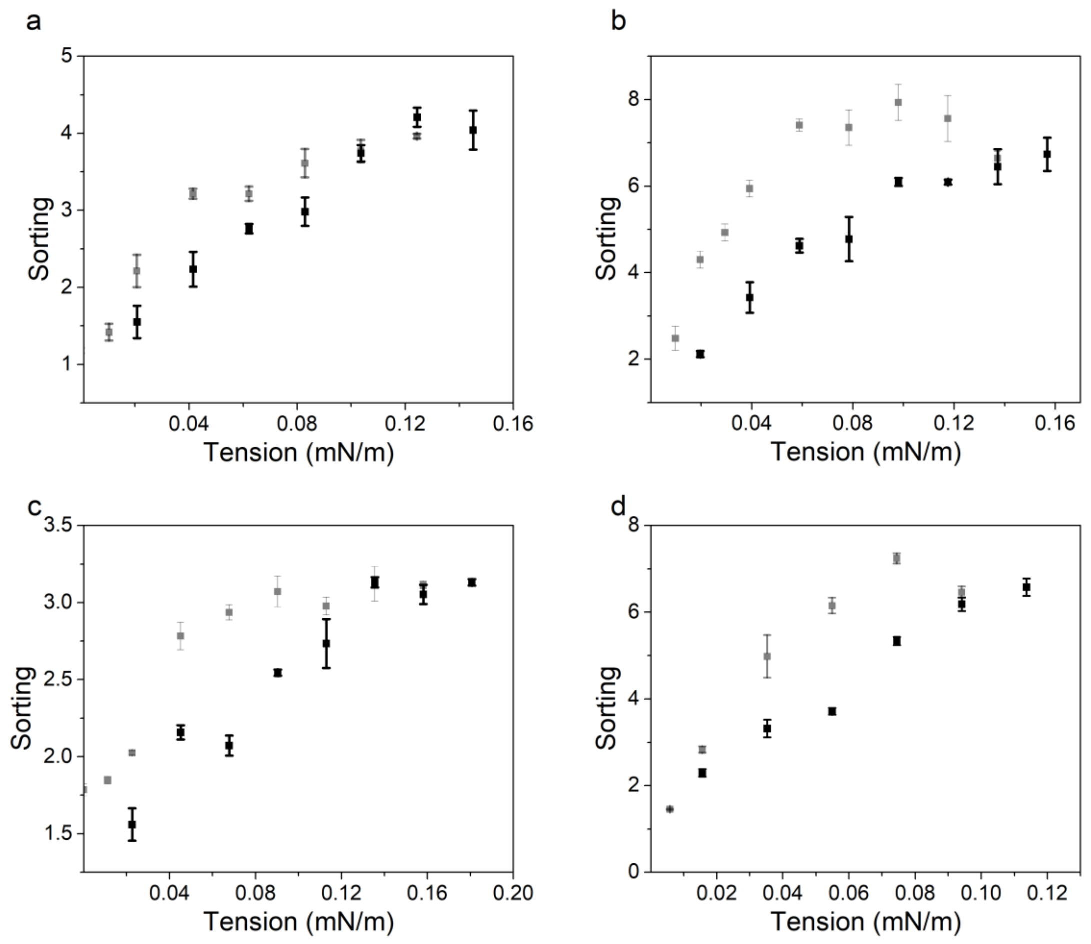

Figure S8. TSPAN4ΔIC tube pulling experiment-tension increase followed by tension decrease. (a-i) Sorting ratio as a function of membrane tension plots of membrane tubes pulled from aspirated GPMVs containing TSPAN4ΔIC-GFP and dyed with DiI-C12. Black and grey squares represent tension increase and tension decrease paths, respectively. Each plot represents a different vesicle.

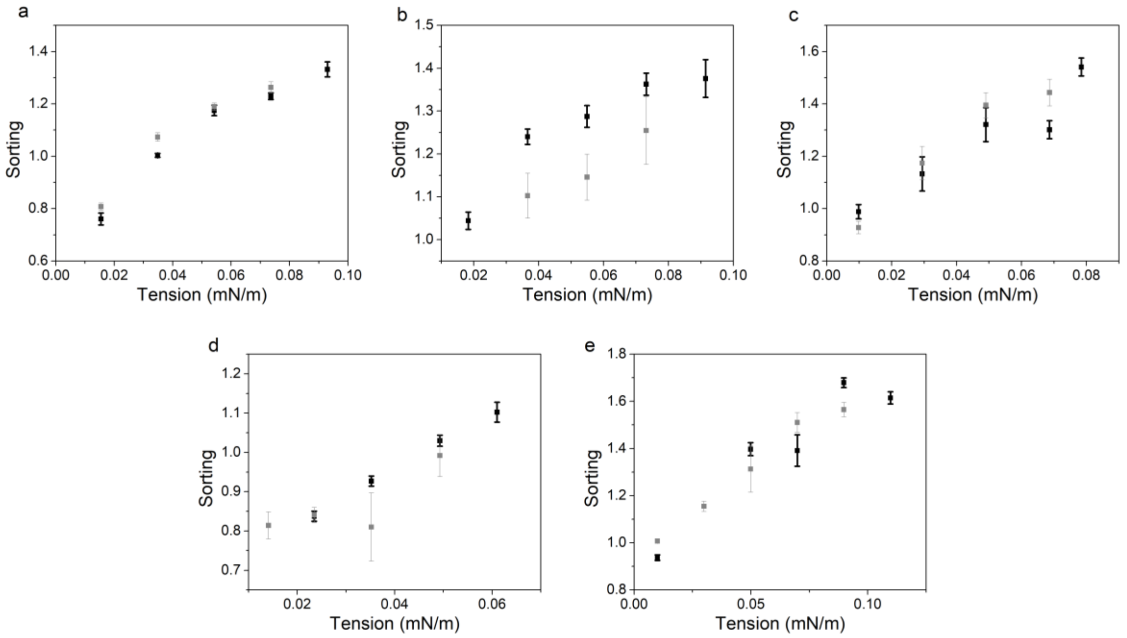

Figure S9. TSPAN4ΔEC2 tube pulling experiment-tension increase followed by tension decrease. (a-i) Sorting ratio as a function of membrane tension plots of membrane tubes pulled from aspirated GPMVs containing TSPAN4ΔEC2-GFP and dyed with DiI-C12. Black and grey squares represent tension increase and tension decrease paths, respectively. Each plot represents a different vesicle.

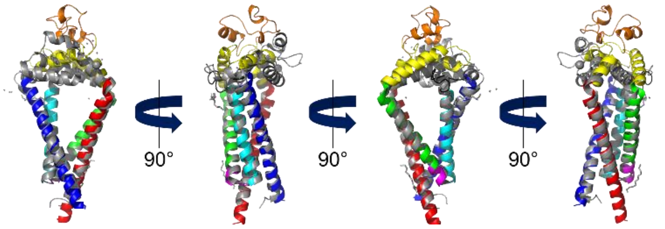

Figure S10. TSPAN4 and CD9 alignment. Alpha fold prediction of TSPAN4 (same color as in Figure1) and crystal structure of CD9 (PDB 6K4J, grey color). The main structural difference between the two proteins is in the EC2 domain (specifically the small loop containing amino acids 151-187 of TSPAN4 which is colored in orange).

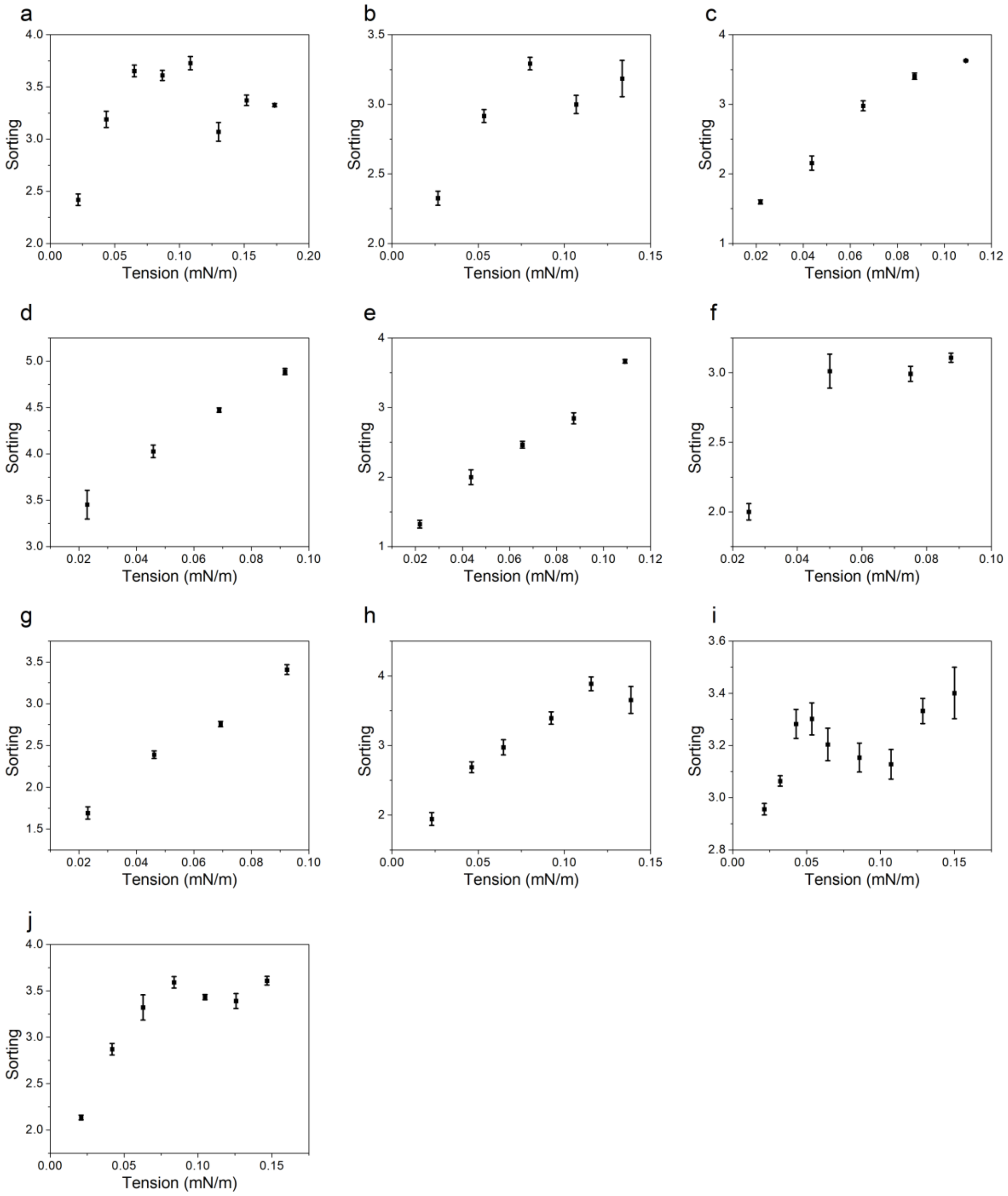

Figure S11. TSPAN4ΔSL tube pulling experiment-tension increase. (a-j) Sorting ratio as a function of membrane tension plots of membrane tubes pulled from aspirated GPMVs containing TSPAN4ΔSL-GFP and dyed with DiI-C12. Each plot represents a different vesicle.

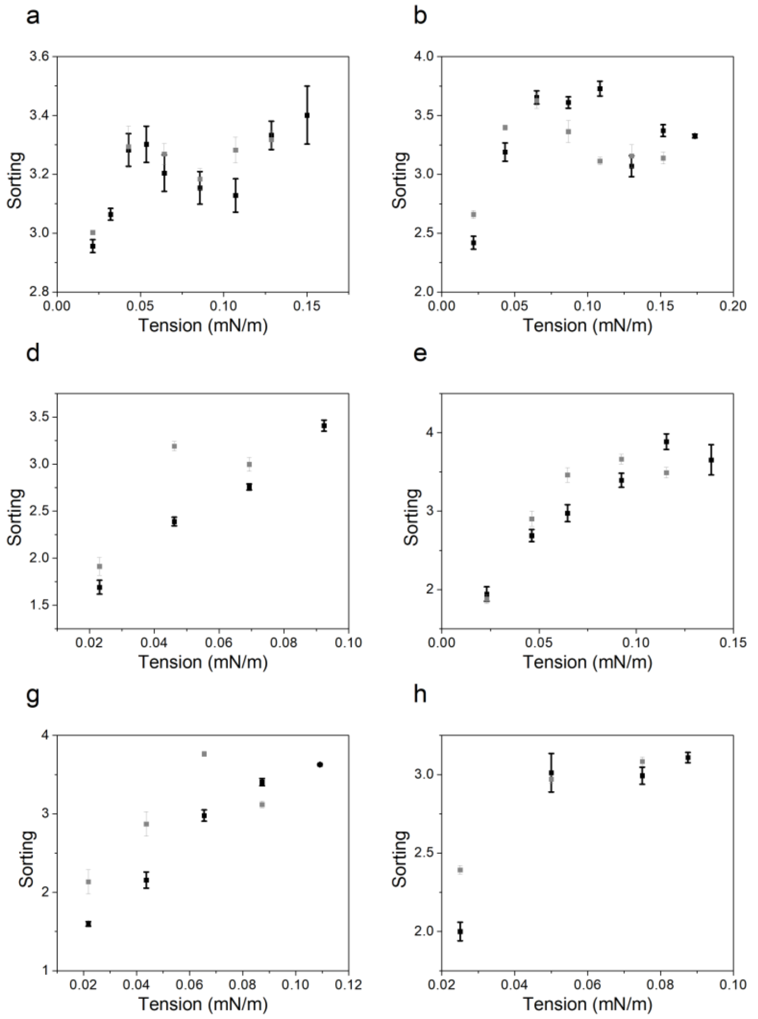

Figure S12. TSPAN4ΔSL tube pulling experiment-tension increase followed by tension decrease. (a-i) Sorting ratio as a function of membrane tension plots of membrane tubes pulled from aspirated GPMVs containing TSPAN4ΔSL-GFP and dyed with DiI-C12. Black and grey squares represent tension increase and tension decrease paths, respectively. Each plot represents a different vesicle.

References:

1. Gerstle, Z., Desai, R. & Veatch, S. L. Giant Plasma Membrane Vesicles: An Experimental Tool for Probing the Effects of Drugs and Other Conditions on Membrane Domain Stability. *Methods Enzymol.* **603**, 129–150 (2018).
